# A high-resolution haplotype collection uncovers somatic hybridization, recombination and intercontinental movement in oat crown rust

**DOI:** 10.1371/journal.pgen.1011493

**Published:** 2024-11-21

**Authors:** Eva C. Henningsen, David Lewis, Eric S. Nazareno, Hayley Mangelson, Monica Sanchez, Kyle Langford, Yung-Fen Huang, Brian J. Steffenson, Brendan Boesen, Shahryar F. Kianian, Ivan Liachko, Eric Stone, Peter N. Dodds, Jana Sperschneider, Melania Figueroa

**Affiliations:** 1 Commonwealth Scientific and Industrial Research Organisation, Australian Capital Territory, Australia; 2 Australian National University, Australian Capital Territory, Australia; 3 University of Minnesota, Saint Paul, Minnesota, United States of America; 4 Phase Genomics, Inc., Seattle, Washington, United States of America; 5 National Taiwan University, Taipei, Taiwan; 6 Cereal Disease Laboratory, USDA-ARS, St. Paul, Minnesota, United States of America; Max Planck Institute for Developmental Biology: Max-Planck-Institut fur Biologie Tubingen, GERMANY

## Abstract

The population structure and evolution of basidiomycetes like rust fungi are influenced by complex reproductive cycles and dikaryotic life stages where two independent nuclear haplotypes are present in the cell. The ability to alternate between asexual (clonal) and sexual reproduction increases the evolutionary capacity in these species. Furthermore, exchange of intact nuclei (somatic hybridization) in rust fungi can allow for rapid generation of genetic variability outside of the sexual cycle. *Puccinia coronata* f. sp. *avenae* (*Pca*), the causal agent of oat crown rust, is a pathogen of global economic importance that is difficult to control due to rapid breakdown of host genetic resistance. The contribution of sexuality, clonality, and migration to virulence evolution varies across *Pca* populations. As such, the *Pca* pathosystem is ideal to address the role of mating type, recombination, mutation, and somatic hybridization in host adaptation. We expanded the existing resources for USA and South African populations by generating whole genome sequencing data of Taiwanese and Australian isolates. An atlas of 30 chromosome-level, fully-phased nuclear haplotypes from six USA isolates and nine Australian isolates was created to capture the genomic composition of key *Pca* lineages. At the haplotype level, we confirmed previous reports of genetic recombination in the USA population and additionally detected either sexual or cryptic recombination between Australian isolates, contrasting previous evaluations that suggested *Pca* populations in Australia to be purely clonal. We also identified somatic hybridization events in *Pca* that are not only associated with significant changes in fitness but also imply intercontinental migration of haplotypes, which provides further impetus for molecular monitoring of rust pathogen populations on a global scale.

## Author summary

Understanding the contribution of different processes to evolution in multinucleated fungi has been challenging due to the genome and life cycle complexity of these organisms. Rust fungi are pathogens of economically important crops and represent organisms in which the interplay between two independent nuclei impacts host adaptation. Recent advances in genomics in various fungal species including *Puccinia coronata* f. sp. *avenae* (*Pca*), the causal agent of oat crown rust, and the characterization of sizeable collections are allowing to finally dissect the contribution of clonality, sexuality, migration and nuclear exchanges to rust evolution. In this study, we generated 30 nuclear genome references for *Pca* isolates from USA and Australia to investigate the genetic diversity of this organism. We also explored the genetic relationship among *Pca* populations from USA, South Africa, Taiwan and Australia. Extensive genetic recombination in the *Pca* population from USA was confirmed and signatures of genetic recombination were also detected in Australian *Pca* isolates. We also found evidence that nuclear exchanges occur in *Pca* and that are associated with changes in host adaptation (virulence). Altogether, our findings make a strong case for monitoring of rust pathogens using molecular tools that allow tracking of individual nuclei.

## Introduction

Understanding the biology and mechanisms contributing to the evolution of Basidiomycete fungi has been difficult due to complex of life cycles and genome structures, multinuclear stages, as well as changes in ploidy. Rust fungi belonging to the group of Basidiomycetes [1,2] exemplify some of these experimental challenges. Nevertheless, the characterization of the genetic and molecular basis of plant immunity was achieved by research on the flax–flax rust (*Melampsora lini*) pathosystem [3–6].

Rust fungi cause many significant agricultural diseases [7], and among these it is worthwhile to highlight the economic impact of cereal rust fungi. Cereal rusts have rapid generation times and long-range air dispersal during the asexual cycle, in which dikaryotic spores, known as urediniospores, repeatedly infect the cereal crop [8,9]. In contrast, the sexual cycle of many cereal rusts only occurs once annually and depends on the availability of an alternate host (other than the cereal crop). Senescence of the cereal host serves as the initial trigger to produce telia, the overwintering structure and first step in sexual reproduction [8]. Teliospores undergo nuclear fusion and meiosis to form haploid spores which infect the alternate host [8,10]. Once mating occurs, the dikaryotic stage of the pathogen is re- established in spores known as aeciospores that can infect the cereal host. Although aeciospores have some dispersal capacity, close spatial co-occurrence of the cereal and alternate hosts is essential to proliferate recombinant genotypes of the pathogen [8,10].

Understanding host adaptation of rust fungi can help supporting disease management strategies and implementation of genomics informed surveillance. Key to this is understanding the genomes of rust fungi, as virulence to host resistance is often a recessive trait in the pathogen and therefore phenotypically silent in heterozygous individuals [6]. However, the full interrogation of dikaryotic genomes was not possible until the development of key technologies like long-read sequencing and haplotype-aware assembly software [11]. The first partial dissection of nuclear haplotypes uncovered high heterozygosity between nuclei of *Puccinia coronata* f. sp. *avenae* (*Pca*—oat crown rust), and *P*. *striiformis* f. sp. *tritici* (*Pst*–wheat stripe rust), highlighting the importance of nuclear phasing to accurately capture genetic variation in dikaryotic species [12,13]. Further improvements in chromatin contact sequencing and phasing pipelines for rusts delivered the first chromosome-level and nuclear phased genomes for *P*. *graminis* f. sp. *tritici* (*Pgt–*wheat stem rust), and *P*. *triticina* (*Pt–*wheat leaf rust) and *Pca* [14–16]. In *Pgt*, this revealed that whole nuclear exchange between strains without genetic recombination had precipitated the emergence of the devastating wheat stem rust Ug99 lineage [14]. Thus, this mechanism for generating diversity enables rapid host adaptation, even in populations without access to the sexual host [9].

Consequently, the paradigm in rust epidemiology has shifted to consider the independent movement of entire haplotypes. This framework was applied to *Pt* where extensive somatic hybridization and intercontinental migration was uncovered through haplotype-level comparative genomics [17]. Nuclear phasing is likewise relevant to understanding the genomes and biology of other fungi. In the basidiomycete mushroom *Tremella fuciformis*, nuclear phasing revealed the formation of new chromosomes following meiosis between asymmetric haplotypes [18]. Similarly, haplotype genomes of the multinucleate fungus *Rhizophagus irregularis* demonstrated that heterokaryotic strains have only two nuclear genotypes [19].

Although molecular resources for studying rust fungi continue to grow, well-established areas of research for other fungi remain poorly characterized in these pathogens. For instance, rusts are known to have mating specificity, but the underlying molecular mechanism governing this is not proven [20]. About 90% of Basidiomycota are heterothallic, meaning that individuals must have different alleles at two loci (*a* and *b*) to undergo sexual reproduction [21]. These loci encode a pheromone and pheromone receptor at the *a* (*PR*) locus and homeodomain-containing transcription factors at the *b* (*HD*) locus [22,23]. The mating loci may be linked (bipolar) or unlinked (tetrapolar). The presence and genomic location of conserved mating type genes in the rust species *Pgt*, *Pt*, and *Pst* suggest heterothallic and tetrapolar systems [20,24]. It has been proposed that mating type also determines somatic compatibility for nuclear exchanges to occur between strains [25].

Oat crown rust disease caused by *Pca* results in significant yield losses worldwide and is difficult to control due to rapid host adaptation by the pathogen [26]. Molecular evidence shows that sexual reproduction is a clear driver of this rapid virulence evolution in USA populations. The sexual host for *Pca* (*Rhamnus cathartica*) is widely distributed in the northern USA but is mostly absent from southern states, so in this case the USA *Pca* population is influenced by both sexual recombination as well as maintenance of clonal lineages [27,28]. Migration within the continental USA facilitates population admixture between these regions [27]. However, asexual *Pca* populations have also been observed to gain virulence following widespread resistance gene deployment [11,26].

To date, only one fully phased, chromosome level assembly (isolate Pca203) and two partially haplotype-separated reference genomes (isolates 12SD80 and 12NC29) are available [12,15], all representing the USA. Given the strong genetic differentiation between USA and South African isolates detected by Hewitt et al [28], the existing references are unlikely to capture the genetic variation of *Pca* globally. Oat crown rust has been detected in Australia since surveys started in the early 1930’s [29], but no references are available. This study combines efforts towards building a haplotype-aware pangenome with the extensive collection of publicly available short-read data for *Pca* to further our understanding of the biology and epidemiology of *Pca* in additional geographic areas through the application of contemporary genomic-based comparative approaches.

## Results

### Contextualization of Australian and Taiwanese *Pca* isolates within a large collection of international isolates

From 2020 to 2023, 137 Australian *Pca* isolates were collected through community submissions across six Australian states and territories (Australian Capital Territory (ACT) = 9, New South Wales (NSW) = 36, Queensland (QLD) = 9, Western Australia (WA) = 55, South Australia (SA) = 10, Victoria (VIC) = 18), and isolates were recovered from both wild (*n* = 111) and cultivated oats (*n* = 26) (S1 Table). Furthermore, four Taiwanese isolates were collected from cultivated oat in 2020 and 2021. Whole genome sequence (WGS) data (Illumina) was obtained from genomic DNA from all isolates (42X average genome coverage; S1 Table). We generated a Maximum Likelihood phylogenetic tree using the WGS data of all isolates and including published data of 211 isolates from the USA, South Africa, and Taiwan (total *n* = 352) [12,15,27,28,30]. Reads were mapped to both haplotypes (average 86% overall mapping, 42% multimapping) of the Pca203 genome reference [15] and filtered variants (*n* = 376,646) were used to construct the Maximum Likelihood phylogenetic tree.

**Fig 1:**
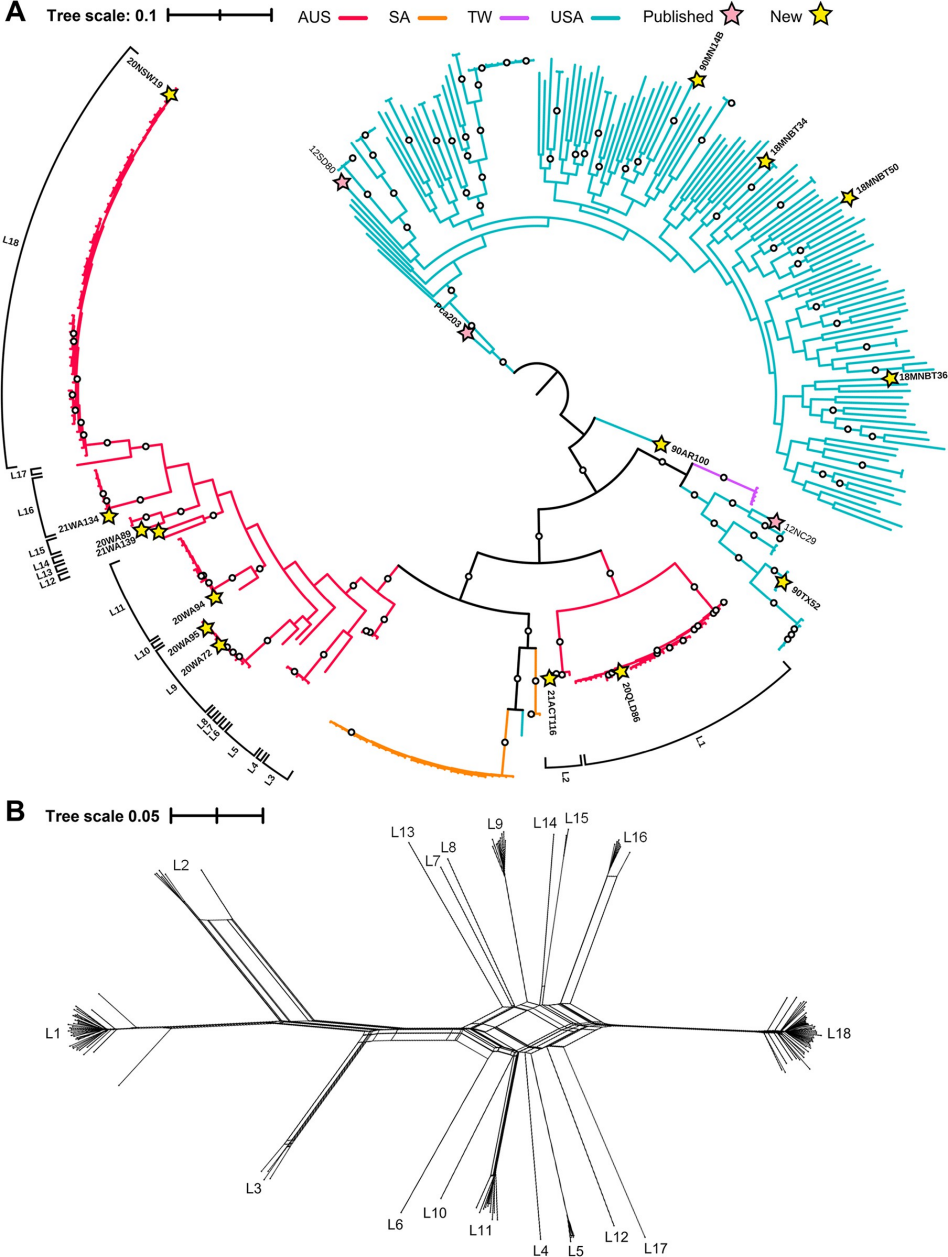
Phylogenetic tree of 352 *Pca* isolates. A) Midpoint rooted Maximum Likelihood phylogenetic tree constructed by mapping reads from 352 *P*. *coronata* f. sp. *avenae* (*Pca*) isolates and calling variants against the full Pca203 reference (hap1, hap2, and unplaced contigs). A total of 376,646 biallelic SNPs and 500 bootstraps were used for this analysis. Tree branches are colored by country of origin: AUS = Australia; SA = South Africa; TW = Taiwan; USA = United States of America. Bootstrap values above 80% shown as white circles. Yellow stars indicate isolates chosen for the haplotype atlas and red stars indicate isolates with existing genome references. Australian lineages L1 through L18 are labelled with brackets. B) Splitstree network of 137 Australian *Pca* isolates from 18 lineages generated from 391,118 SNPs from the entire Pca203 genome. Heterozygous sites were converted to missing. Tree scales are mean substitutions per site.

**Fig 2:**
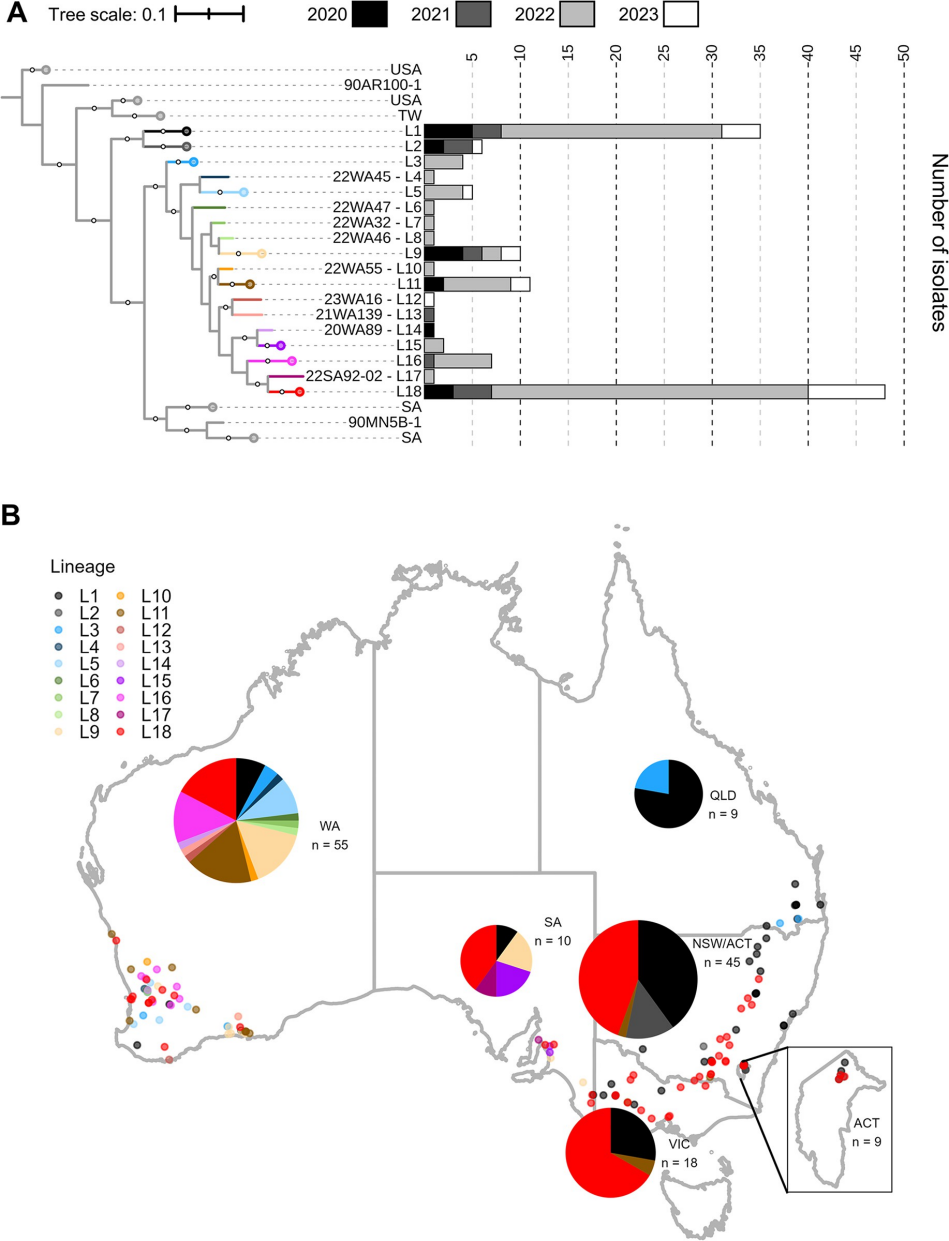
Australian lineage abundance and geographical distribution. A) View of Australian lineages with clones collapsed in the midpoint rooted Maximum Likelihood phylogenetic tree constructed for 352 *P*. *coronata* f. sp. *avenae* (*Pca*) isolates based on 376,646 biallelic SNPs called against the full Pca203 reference (hap1, hap2, and unplaced contigs). Bootstrap values (500 cycles) above 80% shown as white circles. Tree scale is mean substitutions per site. Stacked barplots show the number of isolates of each lineage collected in 2020 (black), 2021 (dark grey), 2022 (light grey), and 2023 (white) across Australian lineages. Tree branches for Australian lineage L1-L18 are colored by lineage to match points and pie charts in panel B. B) Distribution of *Pca* isolates and lineages across Australia. Pie chart sizes reflect a logarithmic transformation of the total number of *Pca* isolates collected in their respective states. The pie chart for NSW/ACT includes isolates from both regions due to the small area of the ACT relative to NSW. Colors reflect lineage assignments.

A total of 18 lineages were detected in the Australian *Pca* collection (Fig 1A), which is unexpected as the population had been proposed to consist of only four asexually-reproducing (clonal) genetic groups [31]. Given this unexpected diversity, we evaluated the entire Australian *Pca* population for recombination by generating a splitstree network (Fig 1B). This analysis showed reticulation between most Australian lineages and the *phi* test (p < 10^−10^) indicated that recombination has occurred to shape the extant Australian population. Although clonality is influential in Australia as shown by the size and temporal persistence of the largest lineages (L18–48 individuals, L1–35 individuals), nine lineages consist of only one isolate. Further, only seven lineages were sampled across multiple years (4 years–L1, L9, L18; 3 years–L2, L11; 2 years–L5, L16) (Fig 2A), suggesting there are more lineages to be characterized. Lineage diversity was greatest in WA with 15 lineages detected (Fig 2B), followed by SA (5 lineages), NSW/ACT (4 lineages), VIC (3 lineages), and QLD (2 lineages). The five most abundant lineages (L1, L3, L9, L11, L18) were sampled on both wild and cultivated oat, indicating that the populations on these different host types are not separate (S1 Table). Only one lineage was detected from the four Taiwanese isolates (Fig 1A), which are all clones of the previously sequenced isolate NTU-01 [30].

To determine the correlation between virulence and lineage assignment based on sequence variation, 95 of the 137 sequenced *Pca* isolates were tested on 27 host genotypes from the Australian oat crown rust differential set, which is commonly used to assign races in surveillance exercises (S1 Fig and S2 Table) [32]. Most isolates in lineage L1 (20ACT25, 22NSW08) displayed virulence across most oat differential lines, although some isolates in this lineage had virulence to fewer lines than the rest (22QLD110, 22NSW107; S1 Fig). Virulence on oat lines Pc63, Pc68, Amagalon (*Pc91*), Culgoa, and WIX4361-9 was unique to L1. Lineage L2 had the least virulence, with 21ACT116 and 23NSW13 only defeating resistance for two lines, Swan (carrying *Pc1*) and Pc70 (also known as H547). The remaining lineages (L3-L18) share many virulence traits, with many isolates having virulence to lines Pc14, Pc45, Pc46, Pc54, Pc61, Pc67, and Pc70 (S1 Fig). Virulence to lines Pc35, Pc36, Pc38, Pc50, Pc52, Pc56, and X716 was rare amongst these lineages. Virulence to lines Pc51 and Pc62 was common in L18 but rare in L3-L17 (S1 Fig). Overall, L1 and L18 are the most broadly virulent lineages, which could explain their prevalence in our sampling. Clustering isolates by these phenotypic results is inconsistent with the phylogenetic relationships (Baker’s Gamma = 0.64, S2 Fig). Thus, race assignment (pathotypes) based on virulence phenotypes are poor predictors of lineage relationships in the Australian *Pca* population.

### Construction of a haplotype collection for *Pca*

From the phylogenetic analysis, we selected 15 representative *Pca* isolates to generate haplotype-phased chromosome-level genome references that capture genetic diversity in Australia (*n* = 9) and the USA (*n* = 6) (S3A and S3B Fig). These included isolates from eight Australian lineages (20QLD86 –L1, 21ACT116 –L2, 20WA72 and 20WA95 –L9, 20WA94 –L11, 21WA139 –L13, 20WA89 –L14, 21WA134 –L16, 20NSW19 –L18), with lineage L9 sampled twice (20WA72, 20WA95) as a baseline for comparing near-identical haplotypes (S3A Fig). The USA *Pca* population experienced a significant shift in virulence between the 1990s and 2010s as reported previously [27]. Thus, we chose three isolates from 1990 (90AR100, 90MN14B, 90TX52; S3A and S3B Fig) with less virulent traits and three broadly virulent, sexually derived isolates from 2018 (18MNBT34, 18MNBT36, 18MNBT50, S3B Fig) [28] to capture diversity before and after the population changed.

**Fig 3:**
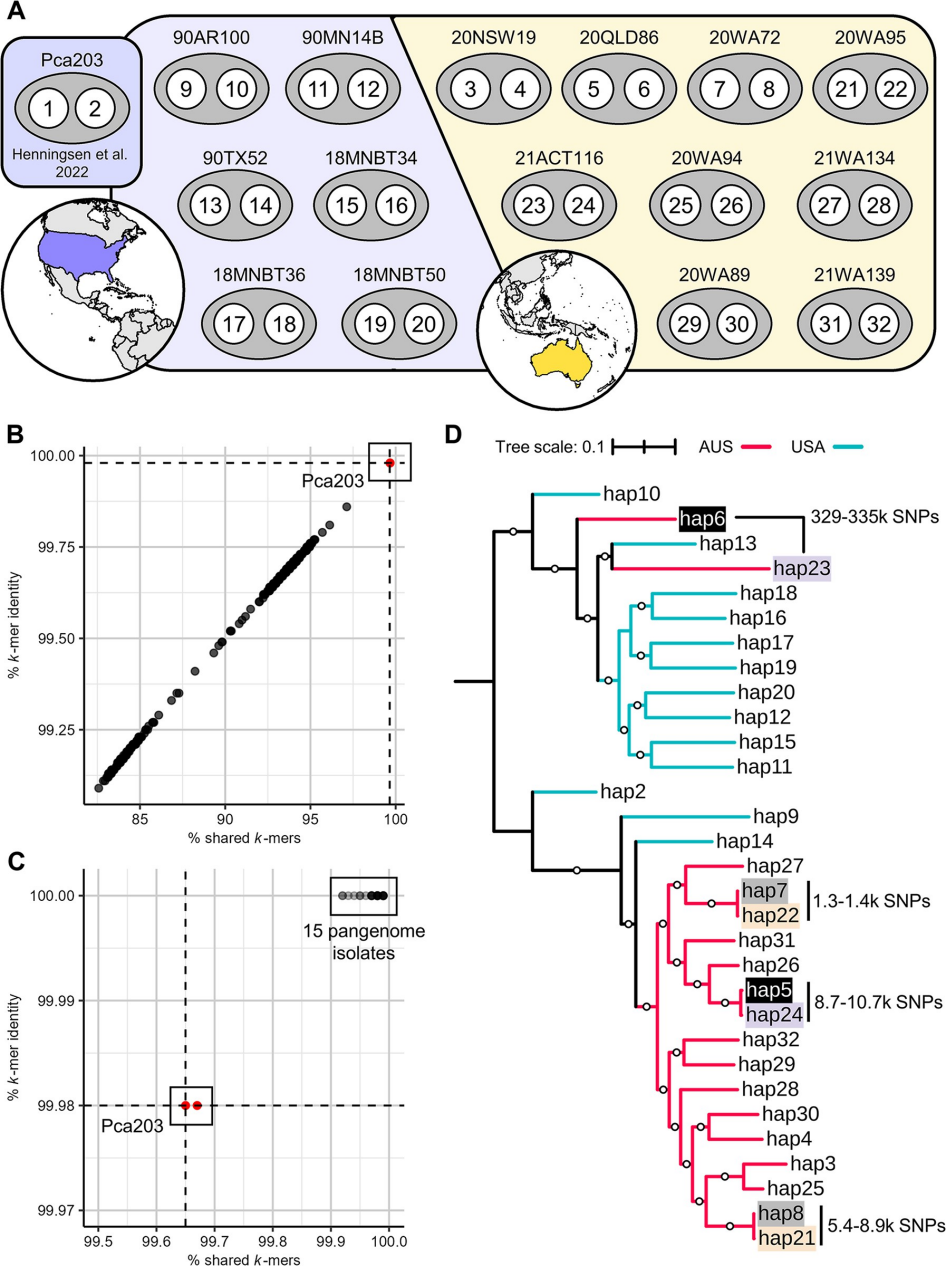
Nuclear haplotypes and sequence comparisons. A) Haplotype numbers assigned to nuclear genotypes of 15 *P*. *coronata* f. sp. *avenae* (*Pca*) isolates from the USA (purple) and Australia (yellow), in addition to previously published haplotypes from Pca203 (1 = “A”; 2 = “B”). B) Plot of percent shared *k*-mers (x-axis) and percent *k*-mer identity (y-axis) obtained by comparing short-read data for 352 *Pca* isolates (points) to hap1 from Pca203. C) Plot of percent shared *k*-mers (x-axis) and percent *k*-mer identity (y-axis) obtained by comparing short-read data for the 16 reference isolates against their haplotypes (i.e. Pca203 reads vs hap1 and hap2, 20NSW19 reads vs hap3 and hap4, etc.). Containment values from Pca203 short reads are highlighted in red. Dashed lines represent thresholds for haplotype containment (≥ 99.98% *k*-mer identity, ≥ 99.65% shared *k*-mers). D) Midpoint rooted Maximum Likelihood phylogenetic tree of 31 *Pca* haplotypes using hap1 as the reference. A total of 219,804 SNPs derived from a pangenome graph alignment were used. Names with matching highlight colors indicate haplotype pairs from the same isolate. Branch colors reflect the country where haplotypes were sampled (AUS = Australia, USA = United States of America). Bootstraps ≥ 80% (500 cycles) are shown as white circles at branch midpoints. Tree scale represents mean substitutions per site.

We generated PacBio-HiFi and Hi-C genome sequence data for these isolates (S3 Table) and assembled nuclear phased genome references following established computational workflows using hifiasm [33] with incorporated Hi-C data [15–17]. This resulted in 30 complete nuclear haplotypes (hap3-hap32; Fig 1A, see yellow stars, Fig 3A and S4 Table). NuclearPhaser [16] detected only five phase swaps in three isolates (90MN14B, 18MNBT36, 20NSW19) that required correction. Four within-haplotype mis-assemblies were also corrected in hap5, hap9, hap10, and hap19. Scaffolding resulted in 18 chromosomes in each haplotype (chr1 to chr18; S4A Fig). An average of 84.44% of *trans* and 96.75% of *cis* and *trans* Hi-C contacts occurred between chromosomes within the same haplotype, confirming accurate haplotype separation and nuclear assignment. *Pca* haplotypes were on average 98.4 Mb long with 57.6% repeat coverage and 19,700 gene annotations, similar to rust fungi like *Pgt* and *Pt* (S4 Table) [14,17]. Haplotype BUSCO completeness averaged 94.85% with less than 2% duplication, which is comparable to haplotype-phased assemblies for *Pgt* and *Pt* (S4 Table) [14,16,17]. The sizes of homologous chromosomes were consistent across haplotypes (average standard deviation = ± 0.21 Mb) except for chr9 (6.389 ± 0.48 Mb; S4A Fig), which harbours a large repetitive region of approximately 0.1 to 1.4 Mb (S4B Fig). To assess the degree of repeat collapsing in this region, the HiFi reads were mapped back onto the main chromosomes. By comparing primary mapping coverage with the average genome-wide coverage, we identified one haplotype (hap31) in which the locus is likely too short in the assembly (S4C Fig). The sharp increase in mapped read coverage at the locus suggests the repeats have been collapsed in hap31. In contrast, the repetitive locus in all other haplotypes has coverage comparable to the genome-wide average which suggests minimal collapsing.

### Characterization of mating loci in the *Pca* nuclear haplotype collection and overall population

To better understand the role of putative *a* and *b* mating type loci in *Pca*, we characterized the *bW-HD1/bE-HD2* and *STE3*.*2* loci using sequences identified in *Pgt* [20]. Pca203 has one copy of *STE3*.*2*.*3* on chr9A and one copy of *STE3*.*2*.*2* on chr9B. Similarly, all isolates included in the *Pca* haplotype collection carry one near-identical copy each of *STE3*.*2*.*2* and *STE3*.*2*.*3* on opposite haplotypes of chromosome 9 (S5A Fig). The *STE3*.*2*.*2* and *STE3*.*2*.*3* alleles are adjacent to the large repetitive region on chr9 which is allele-specific, explaining the unusual chr9 length distribution (S5B and S5C Fig). The *mfa* pheromone precursors were also identified close to these *STE3*.*2* alleles (S5C Fig). The related gene *STE3*.*2*.*1* was invariant and present in a single copy on chr1 in all haplotypes except in hap11 (90MN14B) which had two copies, supporting previous statements that it is unlikely to be involved in mating compatibility [20]. We then characterized the *bW-HD1 bE-HD2* (*HD*) allele pairs across haplotypes. All isolates contained different *HD* alleles in head-to-head orientation in each haplotype on chr4 (S5D Fig) and a total of 13 alleles were recorded (S5E and S5F Fig).

Using these characterized loci, we screened DNA short reads from the entire *Pca* collection (*n* = 352) and found that all isolates contain both *STE3*.*2*.*2* and *STE3*.*2*.*3* alleles (S5 Table) and two different alleles at the *HD* locus (S6 Fig and S6 Table). In *Pca* isolates where one or both *HD* alleles were not characterized in the nuclear haplotype collection, short read mapping still indicates heterozygosity at the *HD* locus (S7 Fig). For populations in Hardy-Weinberg equilibrium, diploid genotypes are expected to occur at specific frequencies depending on the number of alleles present. We used a subset of 88 USA isolates that had been collected as aeciospores from the sexual host (common buckthorn) [28] to test the null hypothesis of genotype equilibrium at the *PR* and *HD* loci in a randomly mating population. The observed genotype frequency for both loci in this subset are 0% homozygous and 100% heterozygous (*PR* locus alleles *n* = 2, *HD* locus alleles *n* = 11), respectively. This is a significant departure from the expected 50:50 (homozygous:heterozygous) ratio for the biallelic *PR* locus in Hardy-Weinberg equilibrium (*p* = 6.55 x 10^−21^). Likewise, the *HD* locus genotype ratio is significantly different than expected (12% homozygous, 88% heterozygous; *p* = 5.70 x 10^−4^). This suggests that both *PR* and *HD* loci contribute to mating type compatibility resulting in exclusive heterozygosity, indicating that *Pca* likely has a tetrapolar mating system as the *PR* and *HD* loci are located on separate chromosomes.

### *Pca* isolates from the USA, Australia, and Taiwan are related through somatic hybridization and migration

To assess the role of somatic hybridization in the evolution of *Pca*, we employed comparative genomics, *k*-mer containment analysis, and phylogenetics approaches, which have been used to identify somatic hybridization events in other rust species [14,17]. We first used variants derived from pangenome graph alignment to generate a Maximum Likelihood phylogenetic tree using hap1 as the reference (Fig 3D). This approach utilizes the nuclear-phased haplotype collection to obtain phased haploid variants at syntenous chromosomal locations, which gives greater resolution than unphased diploid variants obtained by mapping short reads to a reference genome. Three pairs of near-identical haplotypes were identified (hap5 ≈ hap24; hap7 ≈ hap22; hap8 ≈ hap21). Isolates 20WA72 and 20WA95 are clonal members of Australian lineage L9 and as expected their matching haplotypes (hap7 ≈ hap22; hap8 ≈ hap21) were highly similar (1,250–8,913 SNPs; Fig 3D). The other pair of identical haplotypes belong to isolates from different lineages. Hap5 from 20QLD86 (L1) and hap24 from 21ACT116 (L2) were highly similar (8,688–10,667 SNPs). In contrast, their other haplotypes (hap6 and hap23) were extremely dissimilar (328,653–335,515 SNPs). Thus, 20QLD86 and 21ACT116 share only one haplotype, suggesting that they are related through somatic hybridization and nuclear exchange (Fig 3D).

To detect haplotypes shared amongst the broader *Pca* population, we next assessed *k*-mer containment of haplotypes [17] using the short reads from all 352 *Pca* isolates. The lowest containment values for Pca203 haplotypes hap1 and hap2 against its own short read data (99.98% *k*-mer identity, 99.65% shared *k*-mers) were used as the minimum values for identifying shared haplotypes (Fig 3B and S7 Table). The short-read data for the other 15 isolates represented in the haplotype collection exceeded these thresholds when compared to their respective haplotypes (Fig 3C). For example, the short reads for reference isolate 20NSW19 (hap3, hap4) have high containment for hap3 and hap4 (100% *k*-mer identity and ≥ 99.93% shared *k*-mers). As additional evidence to support the *k*-mer containment results (S7 Table), we compared how phylogenetic tree topologies differed when using SNPs from individual haplotypes. As established in *Pgt* and *Pt*, phylogenetic trees constructed from SNPs called against an isolate’s entire genome reference show clonal lineages as discrete clades, while single-haplotype derived trees merge lineages that share the reference haplotype into a single clade [14,17]. Multiple putative nuclear exchange events as detailed below were identified across the entire *Pca* dataset using these criteria.

**Fig 4:**
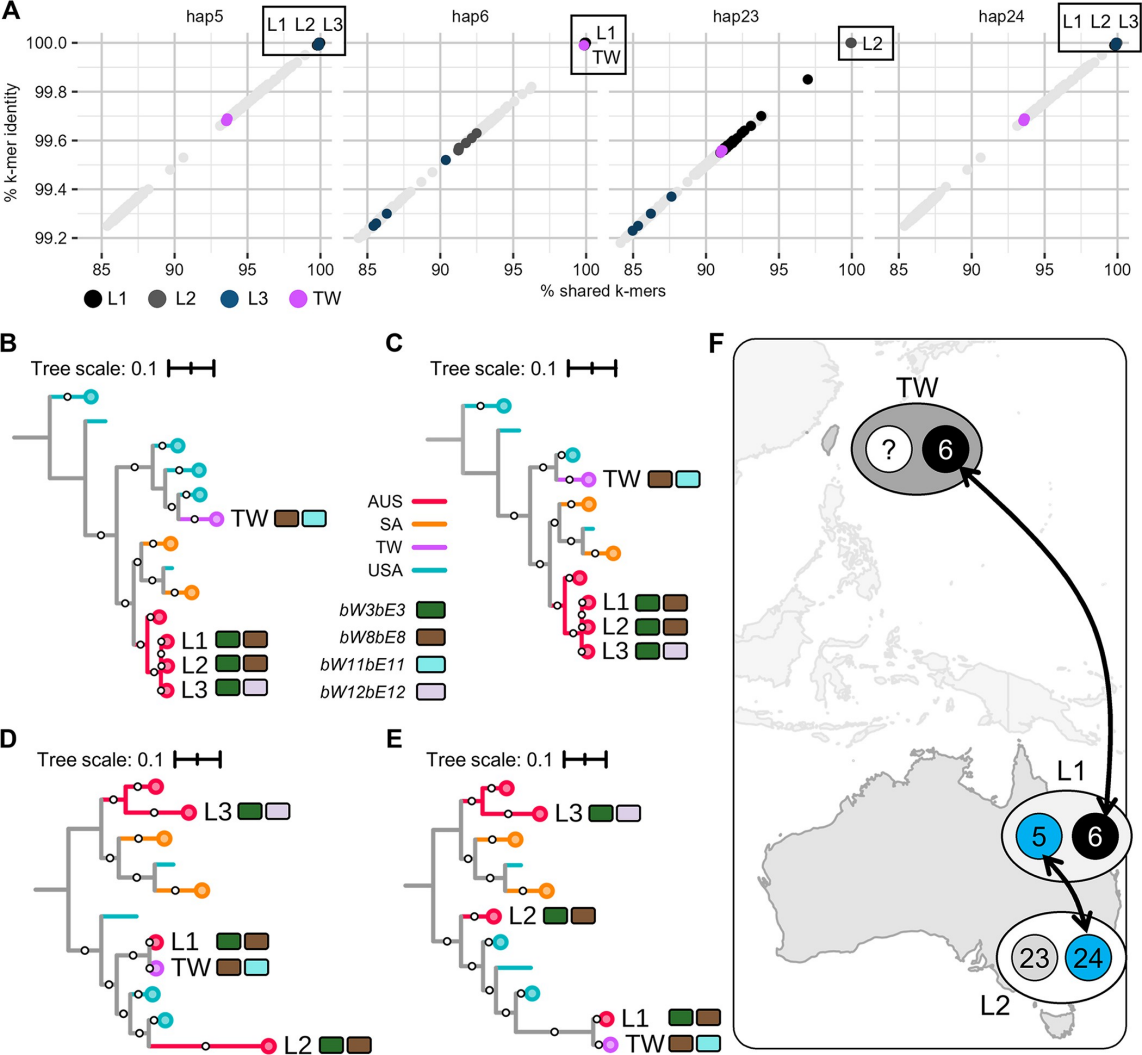
Evidence for somatic hybridization in Australia and Taiwan. A) Plot of percent shared *k*-mers (x-axis) versus percent *k*-mer identity (y-axis) for 352 *P*. *coronata* f. sp. *avenae* (*Pca*) isolates on haplotypes from 20QLD86 (L1; hap5, hap6) and 21ACT116 (L2; hap23, hap24). Colors indicate relevant lineages (L1-L3, TW = Taiwan); light grey points are all other isolates. B-E) Midpoint rooted Maximum Likelihood trees for B) hap5 (225,727 SNPs) C) hap24 (239,896 SNPs) D) hap6 (198,467 SNPs) and E) hap23 (191,316 SNPs). Tree branches are colored by country of origin: AUS = Australia; SA = South Africa; TW = Taiwan; USA = United States of America. 500 bootstraps were generated for each tree and bootstraps 80% (500 cycles) and higher are shown as white circles on branches. Collapsed branches are indicated with circles at leaf tips. Mating type alleles for hybrid lineages are shown as rectangles to the right of branches. Tree scales are mean substitutions per site. F) Proposed relationships between *Pca* lineages and haplotypes overlaid on a map of Oceania and Asia.

The *k*-mer containment results and haplotype-based phylogenies support the proposed somatic hybridization event between a member of L1 and L2 lineages based on full haplotype comparisons. All L1 isolates showed high containment for hap5, hap6, and hap24 (> 99.98% identity, > 99.75% shared *k*-mers), but not hap23 (< 99.86% identity, < 96.99% shared *k*-mers) (Fig 4A and S7 Table). Conversely, all L2 isolates had high containment for hap23, hap24, and hap5 (> 99.98% identity, > 99.82% shared *k*-mers) but not hap6 (< 99.64% identity, < 92.50% shared *k*-mers) (Fig 4A and S7 Table). Phylogenies generated from hap5 and hap24 both show L1 and L2 isolates as members of a single clade, while those for hap6 and hap23 show L1 and L2 as discrete clades (Fig 4B-E). These analyses also suggest another somatic hybridization event linking lineages from Australia and Taiwan, implicating direct or indirect migration of *Pca* (Fig 4F). The five Taiwanese isolates (L-TW) have high *k*-mer containment for hap6 (99.99% identity, > 99.84% shared *k*-mers) but not hap5 (< 99.70% identity, < 93.65% shared *k*-mers) of L1 (Fig 4A and S7 Table). This is also supported by the phylogenetic trees, as L1 and L-TW isolates comprise separate clades in the hap5 tree but cluster together in the hap6 tree (Fig 4B and 4D). Since we did not sample the other haplotype for L-TW in this study, we cannot infer the history of haplotype exchange.

The *k*-mer containment and phylogenetic analyses also support the occurrence of a nuclear exchange within the USA *Pca* population. Members of the 90TX52 lineage (L-1990) each have high containment for both 90TX52 haplotypes, hap13 and hap14 (100% identity, > 99.92% shared *k*-mers) (S8A Fig and S7 Table) as expected. However, another set of seven clonal isolates collected in 2017 (L-2017) from the southern USA (LA, TX, FL) contain hap14 (100% identity, > 99.89% shared *k*-mers), but not hap13 (< 99.85% identity, < 96.65% shared *k*-mers) (S8A Fig and S7 Table). Phylogenetic trees constructed separately from hap13 and hap14 SNPs further support a somatic hybridization event linking L-1990 and L-2017. These lineages appear in separate clades in the hap13 phylogeny (S8B Fig) and as one clade in the hap14 phylogeny (S8C Fig). As the second haplotype of the 2017 isolates was not sampled in the current study, we cannot establish the history of hap14 inheritance (e.g., the other haploid genome donor in the hybridization event) (S8D Fig).

Mating allele composition of putative hybrid isolates agrees with the postulated nuclear exchange events, as somatic hybrid isolates have the same *HD* locus alleles as the haplotypes they are proposed to contain (Fig 4B–4E and S8B and S8C Fig and S6 Table). The assemblies for hap5 and hap24 likewise have the same alleles at both mating loci (*STE3*.*2*.*2* and *bW3bE3*; S5A, S5E, S5F Fig). The other haplotypes do not appear to be related through somatic hybridization events to *Pca* isolates included in the existing genome references.

To assess the role of nuclear exchange on host adaptation in *Pca*, we compared virulence profiles of lineages postulated to be related through somatic hybridization. The virulence of L-TW isolates was previously phenotyped on the USA oat differential set [30], so the subset of 24 resistance sources in common with the 27 lines used in our study were included in comparisons of isolates from L1, L2, and L-TW (S8 Table). Isolates from L1 (hap5, hap6) on average were virulent on 51% of the 24 oat lines. In contrast, isolates of the presumed donor lineages L2 (hap24≈hap5) and L-TW (hap6) on average were only virulent on 10% and 1.7% of these lines, respectively (S8 Table) [30]. L1 isolates had virulence to 14 lines for which no virulence was found in either L2 or L-TW. This suggests that the nuclear exchange event resulted in a substantial change in virulence in the derived lineage. Isolates from L-1990 and L-2017 were previously phenotyped on the full USA oat differential set (S9 Table) [27,28]. A smaller virulence difference is observed between L-1990 and L-2017 isolates, with the average virulence to the 40 lines being 13% for the former and 23% for the latter (S9 Table). Collectively, L-2017 isolates possessed virulence to 11 more differential lines than L-1990 isolates. Altogether, our results support that nuclear exchanges are associated with differences in virulence traits between lineages and contribute to host adaptation.

### The Australian and USA *Pca* populations are shaped by genetic recombination in addition to whole nuclear exchange

**Fig 5:**
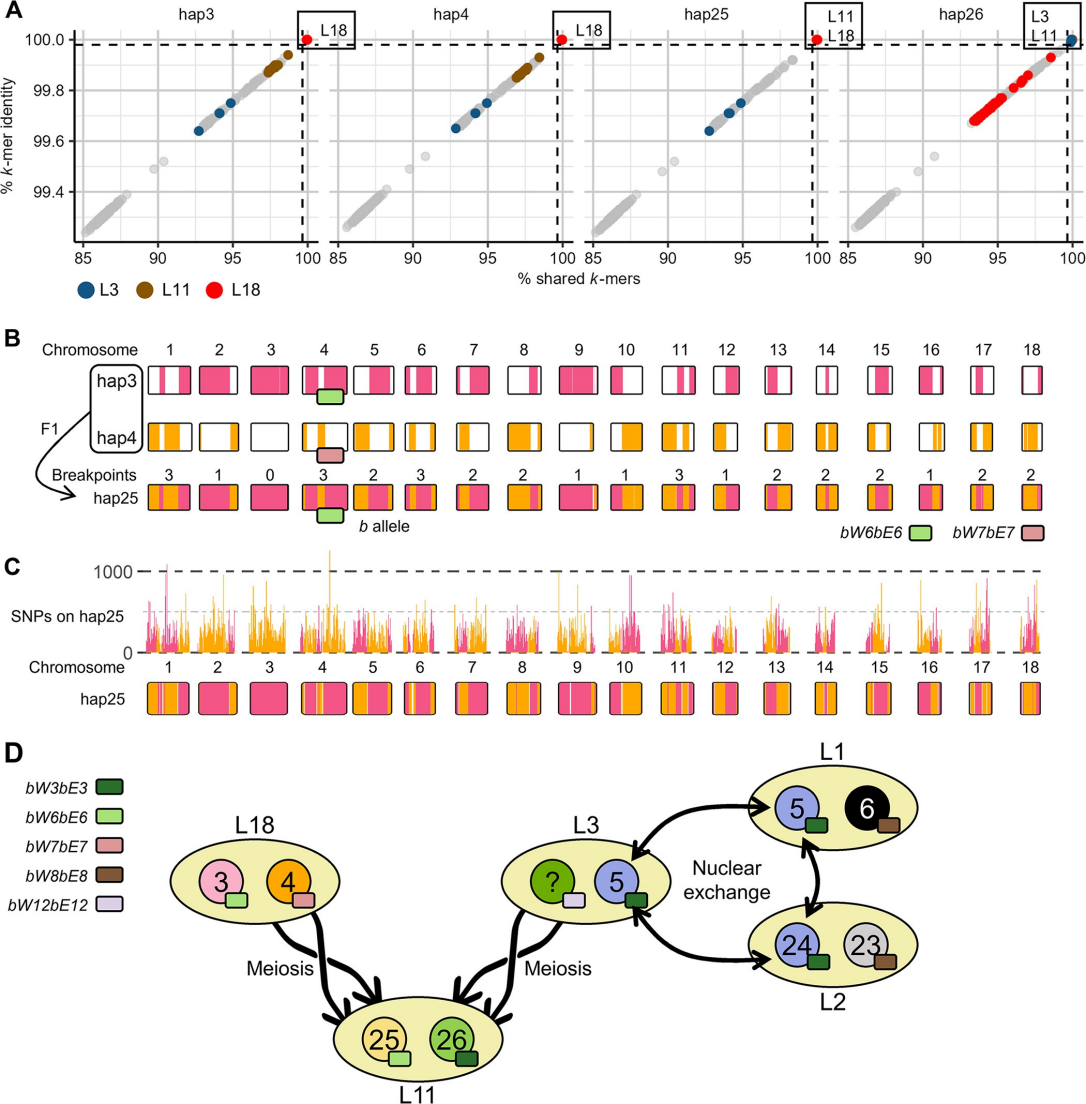
A) Plot of percent shared *k*-mers (x-axis) versus percent *k*-mer identity (y-axis) for 352 *P*. *coronata* f. sp. *avenae* isolates on hap3, hap4, hap25, and hap26. Colors indicate relevant lineages (L3, L11, L18); light grey points are all other isolates. B) Genome alignment between 20NSW19 haplotypes (hap3, hap4) and hap25 from 20WA94. Chromosomes for the three haplotypes are shown with high-identity alignments shown with colored fill (hap3 = pink, hap4 = orange). C) Non-reference variant counts within 100 kb bins from hap3 (pink) and hap4 (orange) on hap25 are shown in the histogram above hap25 chromosomes. Hap25 chromosome fill color is determined by identifying bins with a low density (<50 SNPs/100 kb) of non-reference variants from hap3 (pink) and hap4 (orange). D) Considering the mating type composition and shared sequences between hap5 and hap26, it is possible that hap26 from L11 is the progeny of hap5 and an unknown haplotype from L3. L3 may be related by somatic hybridization to L1 or L2.

The *k*-mer containment analysis also yielded some results that were incompatible with hypotheses of clonality or nuclear exchanges. For instance, the L18 isolates had high containment for hap3 and hap4 from L18 as well as hap25 (100% identity, > 99.91% shared *k*-mers) but not hap26 (< 99.94% identity, < 98.56% shared *k*-mers; Fig 5A and S7 Table) from L11. However, L11 isolates only had high *k*-mer containment for hap25 and hap26 (100% identity, > 99.95% shared *k*-mers) but not for either hap3 or hap4 (< 99.95% identity, < 98.70% shared *k*-mers), as would be expected if L11 and L18 shared a haplotype by somatic hybridization (Fig 5A and S7 Table). Haplotype-specific phylogenetic trees reflected the same relationships, with L18 and L11 isolates forming a single clade in the hap25 phylogeny (S9A Fig) and discrete clades in the hap3, hap4, and hap26 phylogenetic trees (S9B–S9D Fig). Whole-haplotype alignments between hap3, hap4, and hap25 clarified this non-reciprocal relationship. Hap25 contains alternating blocks of high-identity alignments from either hap3 or hap4 covering >95% of this nuclear genome, with 0 to 3 breakpoints between hap3 and hap4 sequences per chromosome (Fig 5B). This is consistent with the frequency of recombination breakpoints in a single meiotic event reported in other rust fungi [34]. Using variants from the pangenome graph of all 32 haplotypes, we examined the distribution of non-reference SNPs from hap3 and hap4 on hap25 and were able to identify the same recombination breakpoints in the whole genome alignments (Fig 5C). This is consistent with hap25 being derived as a haploid product of meiosis from lineage L18 as part of a sexual cross.

We next explored the contradictory finding that L3 isolates had high containment for haplotype hap5 (= hap24) as well as hap26 from L11 isolate 20WA94 (> 99.98% identity, > 99.84% shared *k*-mers; Figs 4A and 5A and S7 Table), despite these haplotypes both having the *bW3bE3 HD* allele pair (S5E–S5F Fig and S6 Table). In support of the haplotype containment results, we found that L3 isolates cluster with L1 and L2 isolates in the hap5 and hap24 phylogenetic trees (Fig 4B and 4C) but cluster with L11 in the hap26 tree (S9B Fig). Lineages L1, L2, L3, and L11 form discrete clades in the hap6, hap23, and hap25 phylogenetic trees (Fig 4D and 4E and S9A Fig). The hap5/24 and hap26 haplotypes all contain the *bW3bE3 HD* allele, while L3 is heterozygous for the *bW3bE3*/*bW12bE12* alleles suggesting some recombination involved in the relationships between these isolates. Alignment of hap5 and hap26 again revealed numerous high-identity segments covering about 45% of haplotype genome in a pattern that suggests recombination (S9E Fig and S10 Table). A model to explain these results is that L3 shares a nucleus (hap5) with lineage L1 and L2 through a nuclear exchange event, while hap26 in L11 is a meiotic recombinant between the two nuclei (hap5 and an unknown haplotype) in L3 (Fig 5D). In this scenario, L11 results from a sexual cross between L3 and L18, which donated the meiotic recombinant haplotypes hap25 and hap26 respectively. The unique sequence amounting to approximately half of the unknown L3 haplotype should also contain *bW12bE12*.

These findings prompted a comprehensive analysis of recombination across the entire *Pca* haplotype collection. We assessed haplotypes from the USA population using the same pangenome graph approach that we validated earlier by comparing parent (hap3, hap4) and F1 (hap25) haplotypes. The numerous small recombination blocks detected reflect frequent recombination between diverse haplotypes in the USA population (S10 Fig), consistent with frequent sexual exchange facilitated by the widespread presence of the alternate host. It is also apparent that the full diversity of the USA population has not been sampled in the haplotype collection, as only 46% of the hap20 sequence is represented by the other 11 USA haplotypes (S10 Fig). USA isolates also display variable degrees of heterozygosity. Isolates like Pca203, 90AR100, 90TX52 have divergent haplotype pairs (1–2% sequence shared), while the other USA isolates are more homozygous (14–16% sequence shared; S10 Table). Hap9 and hap14 have not undergone recent recombination with each other (2.6–2.8% shared) or any other USA haplotypes sampled in our study (0.9–4.0% shared; S10 Fig and S10 Table). In contrast, historic isolate Pca203 clearly had an influential founding effect on the USA population, with hap1 and hap2 contributing from 21.3 to 57.6% of the sequence of other USA haplotypes (S10 Fig and S10 Table).

**Fig 6:**
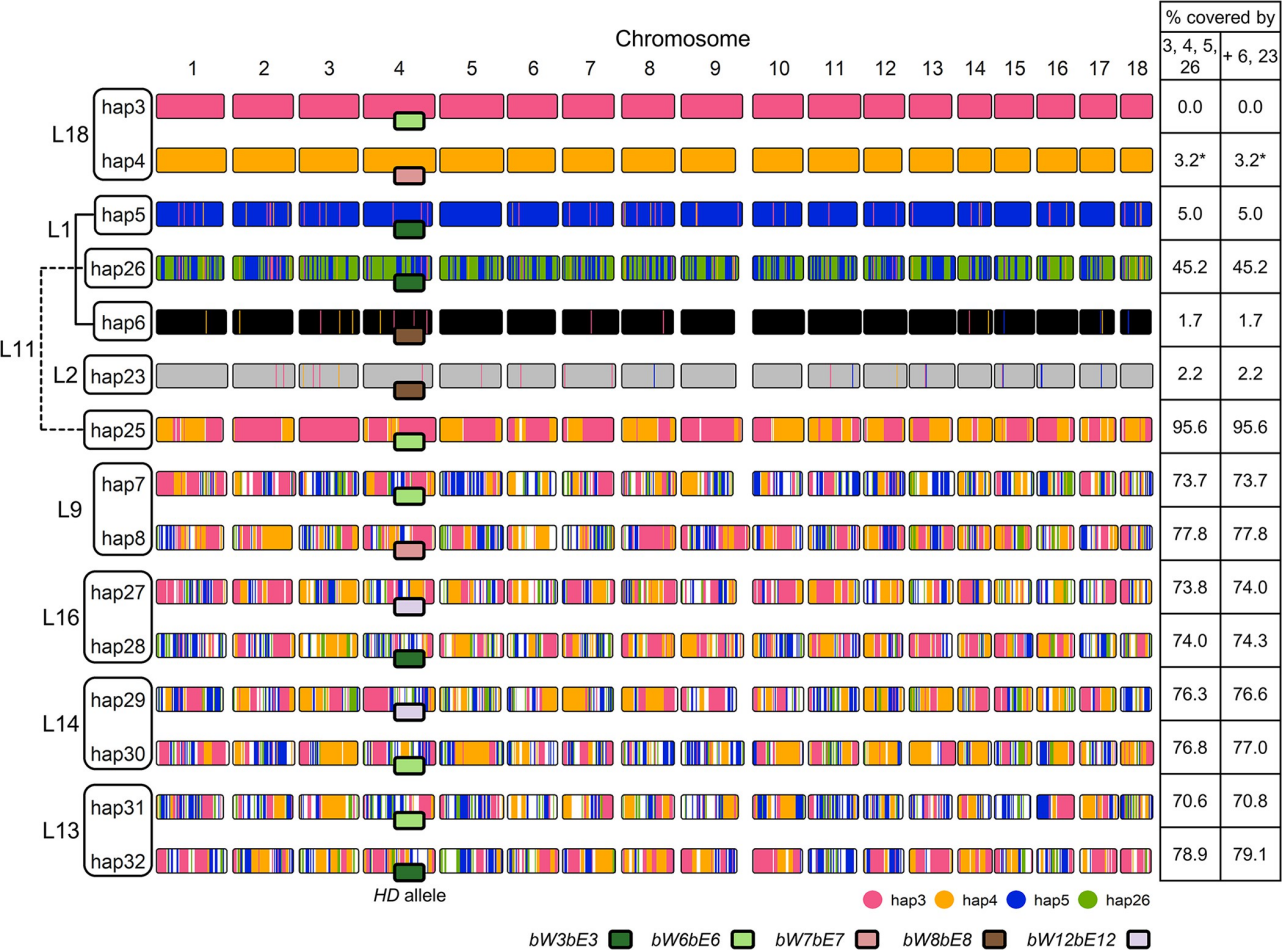
Recombination blocks shared between haplotypes. Shared haplotype blocks between 15 Australian *Pca* haplotypes from founder representatives hap3, hap4, hap5, hap26 and divergent haplotypes hap6 and hap23 identified from 100 kb sequence bins with less than 50 non-reference variants. Shared sequences with hap3 were assigned across the other five haplotypes first, after which the unassigned areas of hap4 were evaluated on the four haplotypes below in the hierarchy, and so on. The haplotype with the shorter name was kept as the representative in cases where haplotypes are nearly identical, as they show redundant information in this analysis (hap8, hap7, and hap5 shown; hap21, hap22, and hap24 not shown). Chromosome fill color represents unassigned regions or regions shared with haplotypes earlier in the hierarchy. Unassigned regions from hap7, hap8, hap27, hap28, hap29, hap30, hap31, and hap32 are filled white. Table on the right side shows percent coverage of each haplotype by those listed in each column. *HD* locus alleles are indicated with fill color of rectangles at the chromosome 4 midpoint. Asterisk (*) indicates that the haplotype blocks on hap4 from hap3 are not visualized for clarity. Raw data is available at: https://github.com/henni164/Pca_pangenome/tree/main/Figure6.

**Fig 7:**
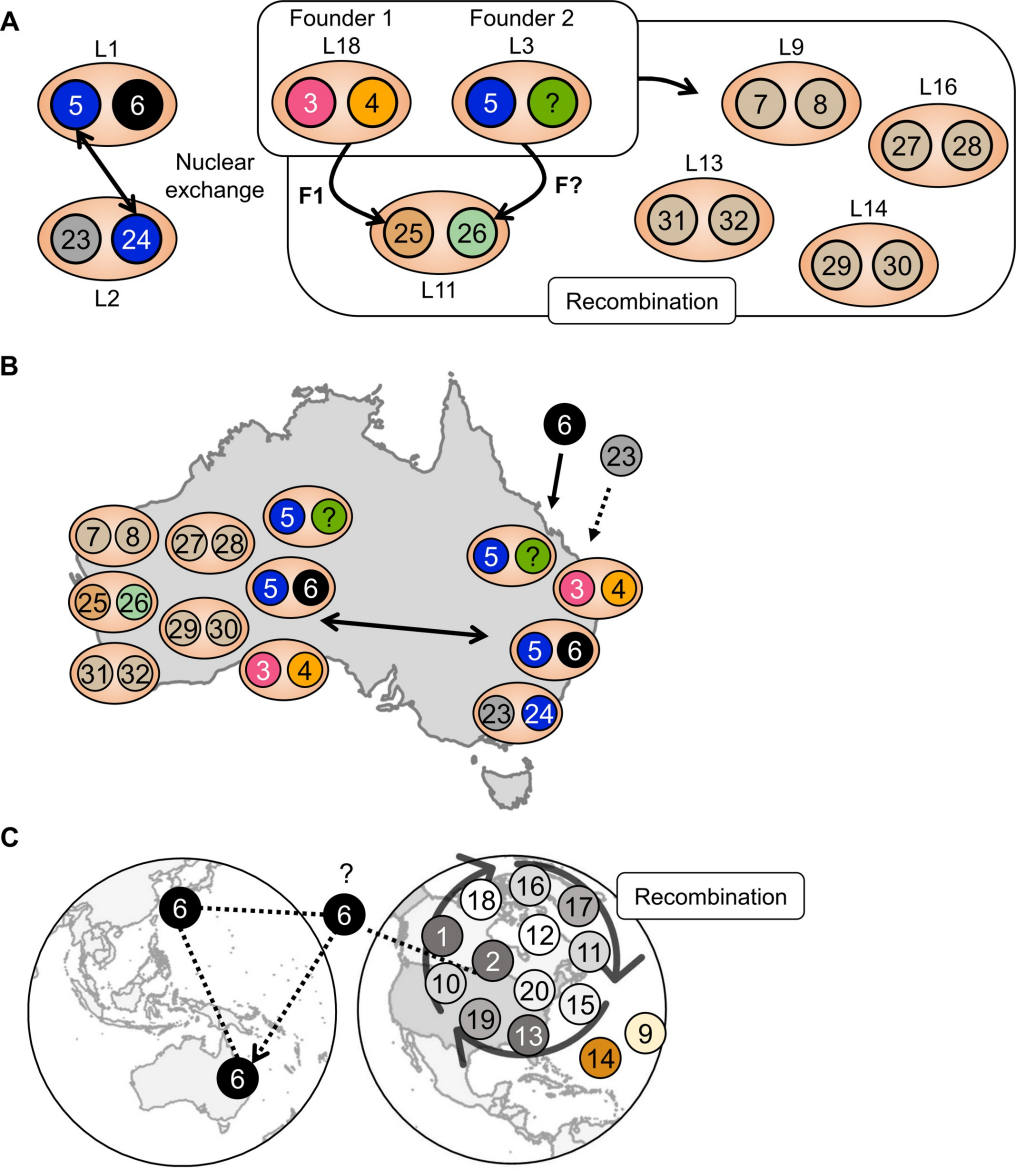
Hypothesized relationships between haplotypes. A) Diagram showing the proposed relationship of haplotypes in the Australian *P*. *coronata* f. sp. *avenae* (*Pca*) population to the two postulated founder lineages L18 and L3. Four colors are assigned to founding haplotypes (pink–hap3, orange–hap4, blue–hap5, green–hap‘unknown’) and derivative haplotypes are color combinations of their ancestors. B) Known distribution of haplotypes in the Australian *Pca* collection in either Western Australia or eastern Australia (Australian Capital Territory, New South Wales, Queensland, South Australia, Victoria). C) Recombination between closely related USA *Pca* haplotypes (shades of grey) and hap6 with simplified possible pathways for hap6 migration.

A similar analysis conducted on the 15 unique Australian haplotypes demonstrates that hap6 and hap23 are divergent from all the others, with almost no shared sequence blocks (Fig 6). However, all the other Australian haplotypes are related through recombination. Both hap3 and hap4 have contributed to all recombinant haplotypes, while hap5 and hap26 have contributed to all except hap25. The large size of recombination blocks between Australian haplotypes relative to those shared between USA haplotypes suggests that recombination is relatively infrequent (Fig 6 and S10 Fig). Australian haplotypes in L3-L18 are approximately 75% covered by hap3, hap4, hap5, and hap26. Considering that hap26 itself is likely derived from meiotic recombination between hap5 and the other unknown haplotype in L3, we hypothesize that the remaining 25% of these haplotypes is present in L3, for which we do not have a genome reference. These data are consistent with the founding of an Australian population consisting of lineages L4-L17 by limited recombination between the four haplotypes represented in the L3 and L18 lineages (Fig 7A). This is further supported by higher levels of homozygosity (19–24% shared) in haplotype pairs from recombinant isolates (20WA72, 20WA89, 21WA134, 21WA139) and the presence of just four *bWbE* locus alleles amongst the L3 to L18 lineages (S7 and S10 Tables). The divergent hap6 and hap23 haplotypes in L1 and L2 have not contributed to the L3-L18 recombining population and given their more restricted geographic distribution, may represent recent incursions (Fig 7B).

There is relatively limited recombination between the haplotypes we sampled from USA and Australian subpopulations, as the total amount of shared sequence blocks between them was generally less than 5% (S10 Table). The only exceptions were hap5, hap6, and hap23 which were 16.7% to 51.5% covered by recombination blocks from the 14 USA haplotypes (S10 Fig). Hap1 and hap2 from Pca203 contribute most to hap6 (31.4%), while the other USA haplotypes share small regions (<5%) with the remaining hap6 sequence (S10 Fig). As hap6 was found in Australia and Taiwan and shares approximately half of its sequence with USA haplotypes, we can infer that intercontinental migration of *Pca* has occurred between North America, Asia, and Australia (Fig 7C).

We assessed the Heaps’ Law model for three types of variation between haplotypes (gene families, shared haplotype blocks, SNPs) to estimate pangenome completeness. Analysis of orthologs from the 29 unique *Pca* haplotypes suggests that most gene families have been captured by the current haplotype collection, as α > 1 (S11A Fig). However, performing this analysis using shared haplotype blocks (S11B Fig) and SNPs (S11C Fig) indicated that further sequence variation remains to be captured (α < 1). It is important to note that the haplotypes in this study form subpopulations (i.e. Australian vs USA haplotypes) with little inter-subpopulation admixture, which violates the assumption of independent sampling. Given that we have not captured the full diversity of the North American population and have not sequenced any isolates from Africa, Asia, Europe, or South America, there is certainly uncharacterized diversity which should be included in future sequencing efforts.

## Discussion

In this study, we investigated the genetic relationships between newly sequenced Australian and Taiwanese *Pca* isolates and previously evaluated populations from the USA and South Africa [12,27,28]. The rich genotypic diversity and dikaryotic lifestyle of this pathogen necessitates the construction of a haplotype-aware pangenome for the species to capture genotypic variability across different geographic areas. We focused on haplotypes from nine Australian and six USA *Pca* isolates as an initial step. This haplotype atlas allowed us to examine mechanisms that could contribute to virulence evolution such as somatic hybridization, migration, mating type, and sexual recombination in *Pca* [9,14,17].

The role of somatic hybridization (nuclear exchange) in the evolution of dikaryotic rust fungi was first proposed in the 1950–1960’s [6,25] and was investigated in *Pca* under controlled conditions [35]. Unfortunately, most early studies assessing somatic hybridization in cereal rust fungi failed to ensure that isolate cross-contamination did not explain detection of new races/virulence profiles. Indisputable proof of nuclear exchange in rust fungi was found by comparing the first chromosome level assembly of wheat stem rust *Pgt*21-0 and sequences from isolate *Pgt* Ug99 [14], which showed that one nuclear haplotype of *Pgt*21-0 is nearly identical to one of the haplotypes of *Pgt* Ug99. The power of haplotype resolution to track the migration and nuclear exchanges shaping global rust populations was subsequently demonstrated for *Pt* [17]. Here we provide evidence that somatic hybridization has also occurred in *Pca* populations in Australia, USA and possibly Taiwan, supporting that nuclear exchanges are common to rust fungi.

Newly acquired and existing whole-genome sequence short-read data and haplotype atlas also enabled population-level *in silico* characterization of mating type control in *Pca*. In other Basidiomycota such as smuts and mushrooms, mating type has been shown to maintain the dikaryotic state, regulate the life cycle, and enforce self/non-self-recognition during mating [36,37]. Although the role of mating loci has not been functionally characterized in the rusts, our finding that all 352 *Pca* individuals in this study are heterozygous at both the *PR* and *HD* loci suggests that mating type controls critical biological functions in *Pca*. Consistent with the findings of Luo et al [38], pheromone precursor *mfa2* is linked closely to *STE3*.*2*.*2* in *Pca*. However, we also identified a 55 amino acid *mfa3* pheromone precursor linked to *STE3*.*2*.*3* which was not reported in their study. Analysis of the *HD* alleles in the haplotype collection generated here support most sequences imputed for *Pca HD* locus alleles published by Luo et al [38]. There is one discrepancy between the *bW5* allele between the studies in which the first 12 N-terminal amino acids of *bW5* defined by Luo et al [38] are not encoded in the haplotypes used to define *bW5* in this study. In addition, the ‘*bW8bE8*’ allele pair reported by Luo et al [38] was only found in a single isolate (90MN5B) that was not included in our haplotype collection. Our study captured three additional alleles (*bW6bE6*, *bW7bE7*, *bW12bE12*) that were not identified before.

Allelic variation at the *HD* locus has been used in rusts like *Pt* and *Pst* to support conclusions regarding migration and population structure derived from genomic and phylogenetic approaches [17,24]. We were able to use the *HD* and *PR* mating type alleles for a similar purpose in our study to support relationships of clonality, recombination, and somatic hybridization. However, several divergent haplotypes (~500-600K SNPs) included in the *Pca* haplotype collection have identical alleles at both mating type loci (i.e., hap6 and hap23, hap3 and hap13, hap5 and hap17). Thus, the mating loci alone are not adequate markers for inferring lineage membership or haplotype composition.

Phylogenetic analysis uncovered 18 lineages in the Australian *Pca* population, in contrast to the *Pca* collections from Taiwan and South Africa that contain only one or two clonal lineages. The numerous lineages found for Australian *Pca* are in stark contrast to other cereal rusts like *Pgt* and *Pt*, which in Australia have only one and five lineages, respectively [14,17]. *Pca* in Australia was likewise presumed to be exclusively clonal based on limited phenotypic information, DNA amplification fingerprinting markers, and the presumed absence of a sexual host [31,39]. However, we found that phenotypic clustering was poorly correlated to genetic lineage structure as has been reported in other *Pca* populations [28]. Further, the evidence of splitstree reticulation between lineages, numerous combinations of *b* locus alleles, and haplotype block exchange instead suggests that recombination between two parental isolates (represented by L3 and L18) founded most Australian *Pca* lineages. While intercontinental migrations could explain these results, the likelihood of F1 haplotypes (hap3, hap4, hap25) migrating together or in separate events is much lower than the hypothesis that rare sexual/parasexual cycles may occur. Together with the evidence of somatic hybridization in L1 and L2, we propose that the extant Australian *Pca* population was derived from four ancestral isolates. Two of these have undergone sexual and/or parasexual processes and the other two donated nuclei via somatic nuclear exchange.

It is yet unclear where or when recombination in Australian *Pca* occurred, and the relative rarity could be explained by sporadic access to a sexual host or uncommon parasexual events. The best-described sexual host for *Pca* (*R*. *cathartica*) is not present in Australia or Oceania. However, other *Rhamnus* species (e.g., *R*. *alaternus*, *R*. *lycioides*, *R*. *palaestina*) have been reported as aecial hosts for *Pca* [26,40,41]. Notably, *R*. *alaternus* was introduced to Australia as an ornamental and is currently managed as an invasive weed [42]. Parasexuality involving anastamosis between two genetically distinct individuals to generate recombinant nuclei is known in Ascomycota like *Verticilium* and may also occur in *Alternaria* [43–45]. A process akin to this has been proposed to occur in rust fungi [46], but this has never been experimentally validated using molecular and genomic tools. The use of field collections in our study means we cannot distinguish between parasexuality or sexuality as the mechanism explaining recombination between Australian haplotypes, as isolates resulting from either somatic recombination or a sexual cross would be genotypically indistinguishable.

Recombination between *Pca* haplotypes from Australia is rare when compared to the haplotypes we sampled from the USA population. Small and frequent recombination blocks among USA haplotypes were detected and are consistent with widespread sexual reproduction facilitated by *R*. *cathartica*, which is prevalent in North America [27,28]. In spite of the high diversity and frequent recombination occurring in the USA *Pca* population, recombination blocks from the historic isolate Pca203 were common in many USA haplotypes. Given that Pca203 (an isolate representing race 203) caused widespread epidemics in the USA in the 1940s [47], it is plausible that Pca203 was an important genetic founder of the contemporary USA population. Pca203 sequence was also prevalent in hap6, which supports migration of *Pca* between Asia, Australia, and North America. Further sampling of haplotypes is needed to improve our understanding of migration and admixture between geographically distant *Pca* subpopulations.

As observed in the USA *Pca* population [27,28], recombination among Australian lineages likely contributes significantly to the high phenotypic diversity in the population [31]. Somatic hybridization also clearly corresponds to significant changes in virulence, as seen in the dramatic differences between the broadly virulent lineage L1 and relatively avirulent lineages L2 and L-TW which are the likely haploid genome donors of the L1 hybrid. As evidenced by variation in virulence within clonal lineages, mutation is also a key influence on virulence evolution. The variation within L1 could support a hypothesis of loss-of-heterozygosity to gain virulence following somatic hybridization, as isolates in this lineage encompass the extremes of restricted to broad virulence. However, the identification and characterization of *Avr* alleles in *Pca* would be necessary to validate this.

Our results suggest that it would be prudent to continue molecular monitoring of *Pca* and investigate the relationship between *R*. *alaternus* or other *Rhamnus* species and *Pca* in the Australian context, as the presence of these invasive species may present significant risk for virulence evolution to the detriment of the oat industry. The haplotype-aware approach to genomics has allowed us to explore the intersection of concurrent evolutionary processes in *Pca* to facilitate virulence evolution. Ongoing efforts to expand this haplotype collection to include members from other continents will further our understanding of the evolution and global movement of *Pca*, as it is clear that we have not captured the entire pangenome of the species.

## Materials and methods

### *Pca* isolates, amplification, and phenotyping

Australian isolates collected from 2020 to 2023 across six states and territories (ACT, NSW, QLD, SA, VIC, WA) have been previously described [32,48] and 75 additional isolates were submitted to CSIRO by community members (see acknowledgements). Isolates from Taiwan (TW) were described previously [30] and are maintained at the National Taiwan University. Australian isolates are maintained at CSIRO (Canberra ACT). Isolates collected in the USA are managed by the USDA ARS Cereal Disease Laboratory in Saint Paul, MN and were previously described [12,15,27,28]. Oat lines for phenotyping were sourced from existing CSIRO seed stocks and the Australian Grains Genebank (AGG) as previously described [32].

Differences between infection procedures for reviving field samples, single pustule purification, and phenotyping are detailed in full in S1 Methods. In all infections, susceptible oat seedlings were grown at 23°C for 16 hours light and 18°C for 8 hours dark. Plants were treated with 15 mL maleic hydrazide per pot at 9 days of growth immediately prior to inoculation. Inoculations were conducted using urediniospores in an oil or talc suspension and infected plants were kept in humidity chambers (90–99% relative humidity) for two days before removal and incubated in growth chambers under the same conditions as before. A subset of 95 isolates were chosen for phenotyping to capture geographic diversity within Australia. For phenotyping, infection types were recorded at 10–11 days post inoculation (dpi), with the final score chosen to reflect the most prevalent infection type across the biological replicates of the same differential line.

### DNA extraction and sequencing

DNA extractions for Illumina sequencing were performed with the G-Biosciences OmniPrep Genomic DNA isolation kit using 20–40 mg of rust spores as input. Libraries were generated using either the Illumina DNA PCR-Free Prep or the IDT Prism library preparation protocol depending on sample needs. Libraries were sequenced to 15-30X depth, 150 bp paired end reads with Illumina Novaseq by Azenta or the Australian Genome Research Facility (AGRF) (S1 Table).

Extractions for high molecular weight (HMW) DNA from rust spores were completed as described previously by Li et al [14]. HMW DNA was used for PacBio HiFi library preparation and sequencing to 30-50X depth at the University of Louisville Sequencing Technology Center or AGRF (S3 Table). Isolates 21ACT116, 20WA94, and 21WA134 were sequenced to 100-200X coverage due to high yield from a single lane of PacBio Revio.

Spores for Hi-C were prepared as described by Sperschneider et al [17] and detailed in S2 Methods. Hi-C libraries were prepared at Phase Genomics, Seattle WA, USA and sequenced with Illumina Novaseq by Azenta Life Sciences (formerly Genewiz) or were prepared and sequenced with Illumina Novaseq at the Ramaciotti Centre for Genomics, NSW, Australia (S2 Methods).

### Genome assembly, phasing, scaffolding, and annotation

Some contamination was apparent in the high-coverage HiFi read data for 20WA89, 21ACT116, and 21WA134 based on large and highly fragmented initial assemblies, so these reads were filtered with mash v2.0 [49] as described in S3 Methods. Raw reads for 20WA94 and 21WA139 and cleaned reads for 20WA89, 21ACT116, and 21WA134 were filtered on length with a 10 kb cutoff and were then randomly down-sampled with seqkit (v2.7.0) sample to 30-50X coverage [50]. PacBio HiFi reads were assembled using Hifiasm with Hi-C integration and resulting contigs were cleaned, phased, scaffolded, and annotated according to [17] and described in detail in S4 Methods. BUSCO values for each haplotype were determined using compleasm v0.2.1 [51] with the odb10 Basidiomycota lineage dataset [52]. In most analyses, unscaffolded contigs are not included as they comprise mostly repetitive sequences which are already represented in the chromosomes (S12 Fig).

### Chromosome 9 coverage analysis

PacBio reads used in the genome assemblies were mapped onto the chromosomes of their respective isolates with minimap2 v2.22 (option—secondary = no) [53]. Coverage was summarised with bbmap v39.06 pileup.sh (sourceforge.net/projects/bbmap/) with binsize = 10000. The ratio of bin coverage to average genomewide coverage was calculated and visualized with R package ggplot2 v3.5.1 [54].

### Identification of putative mating type genes and population screening

Protein sequences for *STE3*.*2*.*1*, *STE3*.*2*.*2*, *STE3*.*2*.*3*, *bW-HD1* and *bE-HD2* from *Pgt* were BLASTed (tblastn) against the Pca203 genome reference with BLAST+ v2.13.0 [15,20,55]. Proteins overlapping the best hits were compared to the *Pgt* alleles and examined for conserved domains known to be present and intron/exon count before use in searching the other 30 haplotypes (S4 Methods) [20,21]. Protein sequences for *STE3*.*2*, *bW-HD1*, and *bE-HD2* alleles were aligned with CLUSTALW, and phylogenetic trees were constructed with the built-in ‘raxml-bootstrap’ option [56]. Protein trees were rooted on alleles from *Pgt* CRL 75-36-700-3 (*Pgt STE3*.*2*.*1* and *Pgt bW-HD1*) with R package ‘ape’ and visualized with iTOL [20,57,58].

To screen the *Pca* population for the presence of the *STE3*.*2* and *HD* alleles, the 3’ to 3’ DNA sequence of the *HD* locus and 5’ to 3’ sequences of *STE3*.*2*.*2* and *STE3*.*2*.*3* were extracted from each haplotype. These loci were sketched with mash v2.0 (-s 1000) [49] and then screened with the Illumina reads for 352 isolates (S1 Table) [12,15,27,28,30]. Alleles were considered contained at or above 99% *k*-mer identity and 94% shared *k*-mers.

To compare the repetitive regions on chromosome 9, haplotypes were aligned with minimap2 v2.22 (-k19 -w19 -m200 -DP -r1000) and visualized with ggplot2 v3.5.1 [53,54].

### Population screening for haplotype containment

The 352 *Pca* isolates with Illumina data (S1 Table) were screened against the 32 available *Pca* haplotypes using mash v2.0 [49]. These haplotypes were processed with mash sketch (-s 100000) and mash screen was run comparing the Illumina data against the haplotype sketch file. Isolates with *k*-mer identity of ≥ 99.98% and shared *k*-mers ≥ 99.65% were considered as likely containing the screened haplotype based on the lowest containment of Pca203 haplotypes (hap1, hap2) by Pca203 short reads. Candidate hybrids were identified for having high identity to only one haplotype, or as having high identity to two haplotypes which were not contained within the same reference isolate.

### Phylogenetic analysis

Illumina reads for 352 isolates (S1 Table) were mapped to the diploid assemblies for isolates Pca203, 90TX52, 20NSW19, 20QLD86, 20WA94, and 21ACT116 using bwa-mem2 v2.2.1 [59]. Variants were called against the diploid assembly of Pca203 and individual haplotypes (3, 4, 5, 6, 13, 14, 23, 24, 25, 26), using freebayes v1.3.6 (—use-best-n-alleles 6) [60]. Variants were filtered with vcflib v1.0.1 (-f "QUAL > 20 & QUAL / AO > 10 & SAF > 0 & SAR > 0 & RPR > 1 & RPL > 1 & AC > 0") and vcftools v0.1.16 (—min-alleles 2—max-alleles 2—max-missing 0.9—maf 0.05) [61,62]. Variants were converted to PHYLIP format using vcf2phylip [63]. The missing call threshold removes approximately 1/3 of the filtered SNPs (using Pca203 as the reference, 364,744 of the 1,081,096 filtered SNPs were removed). RAxML v8.2.12 was used to construct the Maximum Likelihood tree with 500 bootstraps (-f a -m GTRCAT -# 500—no-bfgs) [64]. The resulting Maximum Likelihood tree was visualized with iTOL [58].

Variants called against the Pca203 complete reference for the 137 Australian isolates were filtered to include only biallelic single-nucleotide polymorphisms (SNPs). Variants were converted to PHYLIP format with vcf2phylip [63], and heterozygous calls were converted to missing (N) resulting in 391,118 SNPs. Splitstree CE v.6.2.1-beta was used to calculate Hamming distances and generate a neighbor net with 301 splits (https://github.com/husonlab/splitstree6) [65]. The network was evaluated with the phi test for recombination in Splitstree CE v6.2.1-beta [66].

### Comparison of pathotype clustering and phylogenetic tree structures

Infection types were converted to a binary system of 0 (avirulent, infection types 0 to 2) and 1 (virulent, infection types 3 to 4) and clustered with R v4.3.2 hclust() before conversion to a dendrogram [67]. The Pca203 Maximum Likelihood tree was pruned to contain only phenotyped Australian isolates with iTOL [58] and was modified to be binary and ultrametric with R package ‘ape’ before conversion to a dendrogram [57]. The two dendrograms were compared with untangle_step_rotate_1side() from ‘dendextend’, with the phenotype clustering dendrogram being rotated to find the best structural match to the RAxML-derived dendrogram [68]. The comparison was visualized with tanglegram(rank_branches = TRUE) from ‘dendextend’ to visualize topology without considering branch lengths [68]. Finally, the two dendrograms were compared with cor_bakers_gamma() from ‘dendextend’ to calculate the correlation between the tree structures [68].

### Haplotype comparisons

Haplotypes (hap3 and hap4 versus hap25; hap5 versus hap26) were aligned with D-Genies v1.5.0 and visualized with ggplot2 v3.5.1 [54,69]. The cactus-pangenome pipeline from cactus v2.6.6 was run on all haplotypes and variants were called for each haplotype as the reference [70]. For the phylogenetic tree of haplotypes, variants against hap1 were converted to PHYLIP format with vcf2phylip [63]. RAxML v8.2.12 was used to construct the Maximum Likelihood tree with 500 bootstraps (-f a -m GTRCAT -# 500—no-bfgs) [64] and the resulting Maximum Likelihood tree was visualized with iTOL. [58]. Based on published comparisons between *Pt* haplotypes [17], *Pca* haplotype pairs that were less than ~0.01% diverged (~10,000 SNPs in 100 Mb haplotype) were considered near identical.

The full procedure for determining shared haplotype blocks is detailed in S5 Methods. Briefly, variant counts were binned and a cutoff of < = 50 non-reference SNPs per 100kb bin was applied. Adjacent shared regions for each sample-reference pair were merged, subjected to an ordered bedtools (v2.31.1) [71] subtraction, and regions < = 50 Kb were removed before visualization.

### Estimating pangenome completeness

Orthofinder v2.5.4 [72] was run on all proteins on chromosomes of unique haplotypes generated in this study and published proteins from Pca203 (https://doi.org/10.25919/fdb7-sc82) [15]. The orthogroup counts and unassigned genes outputs were merged and used as the presence/absence input for R package micropan v2.1 [73]. Recombination block assignments from the pipeline described above were converted to a matrix of presence/absence values. Finally, the SNPs against hap1 from the cactus-pangenome pipeline were processed in a similar manner, with reference SNPs being treated as “absence” and non-reference SNPs as “presence”. micropan function heaps() was used to estimate the Heaps’ Law decay parameter α and function rarefaction() was used to generate random permutations (n = 1000) of cumulative counts for orthogroups, haplotype blocks, and SNPs.

### Map visualization

The map of Australia with state borders was drawn using R package ‘ozmaps’ v0.4.5 [74], which uses the 2016 Local Government Area data from the Australian Bureau of Statistics [75]. The globe-projected world maps were drawn using R package ‘giscoR’ v0.5.0 [76] with GISCO map data released by Eurostat. Flat world maps were constructed with R ‘maps’ v3.4.2 [77], which uses public domain data from the Natural Earth project (https://www.naturalearthdata.com/).

## Supporting information

S1 Fig Phylogenetic tree and phenotypes of 18 Australian lineages. Midpoint rooted Maximum Likelihood phylogenetic tree constructed by mapping reads from 352 *P*. *coronata* f. sp. *avenae* (*Pca*) isolates and calling variants against the full Pca203 reference (hap1, hap2, and unplaced contigs). 376,646 biallelic SNPs and 500 bootstraps were used. Tree branches are colored by country of origin: AUS = Australia; SA = South Africa; TW = Taiwan; USA = United States of America. Bootstrap values (500 cycles) are percentages (100 = 100%). Tree scale is mean substitutions per site. Heatmap represents isolate virulence on the differential lines (virulent = red, avirulent = yellow). Isolates that were not phenotyped were pruned from the tree, resulting in the removal of L7 and L17.
(TIFF)

S2 Fig Comparison of tree topologies. Comparison between tree topologies of 16 Australian *P*. *coronata* f. sp. *avenae* (*Pca*) lineages from clustering by pathotype (left) versus phylogenetic relationships (right). The Maximum Likelihood tree (right) was generated with 376,646 SNPs from 352 *Pca* isolates against the complete Pca203 genome (hap1, and hap2, and unplaced contigs), which was pruned to contain only phenotyped Australian isolates and midpoint rooted. The R package ‘tanglegram’ was used to rotate the pathotype clustering tree branches until the best match to the phylogenetic tree was found. Branch lengths are arbitrary in the visualization and assessment of similarity. Solid black lines in the tree structure indicate edges found in both trees. Lines in the center connect the same isolate across trees, with colored lines showing clusters containing more than one isolate which are structurally identical.
(TIFF)

S3 Fig Reference isolates in phylogenetic tree. Midpoint rooted Maximum Likelihood phylogenetic tree of 352 *P*. *coronata* f. sp. *avenae* (*Pca*) isolates constructed by mapping reads and calling variants against the full Pca203 reference (hap1, hap2, and unplaced contigs). 376,646 biallelic SNPs and 500 bootstraps were used. A) Collapsed tree view of primarily Australian lineages (L1 to L18). Except for Pca203, the first two digits of the name of the *Pca* isolate reflect year of collection, followed by state and sample identifier. B) Collapsed tree view showing USA lineages. USA *Pca* lineages were not numbered as the population is highly diverse. Tree branches are colored by country of origin: AUS = Australia; SA = South Africa; TW = Taiwan; USA = United States of America. Bootstrap values above 80% shown as white circles. Yellow labels indicate isolates chosen for the haplotype atlas and red labels indicate isolates with published references. Tree scales are mean substitutions per site.
(TIFF)

S4 Fig Chromosome lengths and analysis of repetitive chr9 locus. A) Boxplots of chromosome lengths for the 32 *P*. *coronata* f. sp. *avenae* (*Pca*) haplotypes with black dots indicating chromosome size of each haplotype. Box top and bottom boundaries are the upper and lower quartiles, respectively. Lines within boxes represent the mean. Lines extending below and above boxes delimit minimum and maximum values. B) In descending order: alignments of chromosome 9 regions containing STE3.2 genes between hap13 and hap25 (*STE3*.*2*.*2*), hap26 and hap27 (*STE3*.*2*.*3*), and hap13 and hap26 containing different *STE3*.*2* alleles. C) Ratio between HiFi read coverage within 10 Kb bins and average genome wide coverage on chromosome 9 across four *Pca* haplotypes (hap25, hap26, hap31, hap32). Chromatin contact maps of chromosome 9 for each haplotype are shown to the right, with colors representing contact frequency (red = high, blue = low).
(TIFF)

S5 Fig *PR* and *HD* mating locus information. A) Phylogenetic tree of *STE3*.*2* alleles in 32 *Pca* haplotypes, rooted at *P*. *graminis* f. sp. *tritici STE3*.*2*.*1*. B) Boxplots showing chromosome 9 length distribution separated by which *STE3*.*2* allele is present. Box top and bottom boundaries are the upper and lower quartiles, respectively. Lines within boxes represent the mean. Lines extending below and above boxes delimit minimum and maximum values. C) Orientation and arrangement of *STE3*.*2* and *mfa* (*PR*) alleles on chromosome 9 (chr9) of hap25 and hap26 from *P*. *coronata* f. sp. *avenae* (*Pca*) isolate 20WA94. D) Orientation and arrangement of *HD* alleles on chromosome 4 (chr4) of hap25 and hap26 from *Pca* isolate 20WA94. E-F) Midpoint-rooted phylogenetic trees of E) *bW-HD1* alleles and F) *bE-HD2* alleles in 32 *Pca* haplotypes. Colors indicate identical *HD* alleles within each tree and allele pairs across trees (i.e. *bW3* and *bE3* are always found in the same haplotype), as recombination between *bW* and *bE* was not detected in our haplotype atlas. Branches with bootstraps (100 cycles) over 80% are shown with white circles at the midpoint. Tree scales are mean substitutions per site.
(TIFF)

S6 Fig Phylogenetic tree with *HD* alleles. Midpoint rooted phylogenetic tree constructed by mapping short reads from 352 *P*. *coronata* f. sp. *avenae* isolates and calling variants against the complete Pca203 genome (hap1, hap2, unplaced contigs). 376,646 biallelic SNPs were used over 500 bootstraps to produce the Maximum Likelihood tree. Bootstrap values are percentages (100 = 100%). *HD* alleles are shown as rectangles next to branches. Tree branches are colored by country of origin: AUS = Australia; SA = South Africa; TW = Taiwan; USA = United States of America. Tree scales are mean substitutions per site.
(TIFF)

S7 Fig *HD* locus variants without two defined alleles. Variants called for 49 *P*. *coronata* f. sp. *avenae* isolates with one or two unknown *HD* locus alleles in the *HD* locus regions of Pca203 hap1 (*bW1bE1*) and hap2 (*bW2bE2*). Line colors indicate missing (white), reference (black) and alternative (pink, green, blue, orange, yellow) genotypes. Half-length lines indicate heterozygous sites and full-length lines indicate homozygous sites.
(TIFF)

S8 Fig Evidence for somatic hybridization in the USA. A) Plot of % *k*-mer identity (y-axis) versus % shared *k*-mers (x-axis) for short reads from 352 *P*. *coronata* f. sp. *avenae* isolates compared to hap13 and hap14. Colors indicate relevant lineages; light grey points are all other isolates. Midpoint rooted Maximum Likelihood phylogenetic trees constructed from variants from individual haplotypes B) hap13 (194,540 SNPs), C) hap14 (209,542 SNPs) with bootstraps 80% or higher (500 cycles) shown as circles at branch midpoints. Tree branches are colored by country of origin: AUS = Australia; SA = South Africa; TW = Taiwan; USA = United States of America. Collapsed clades are indicated by circles at tips. *HD* alleles are shown as colored rectangles next to relevant clonal lineages. Tree scales are mean substitutions per site. D) Diagram of the proposed relationship between USA lineages and haplotypes involved in somatic hybridization.
(TIFF)

S9 Fig Evidence for recombination connecting between Australian lineages. Midpoint rooted Maximum Likelihood phylogenetic trees for 352 *P*. *coronata* f. sp. *avenae* isolates constructed from variants from individual haplotypes A) hap25 (205,993 SNPs) B) hap26 (188,803 SNPs) C) hap3 (228,300 SNPs) D) hap4 (226,617 SNPs). Bootstrap supports 80% or higher (500 cycles) are shown as circles at branch midpoints. Tree branches are colored by country of origin: AUS = Australia; SA = South Africa; TW = Taiwan; USA = United States of America. Collapsed branches are indicated by circles at tips. *HD* alleles are shown as colored rectangles next to relevant clonal lineages. Tree scales are mean substitutions per site. E) Regions of 20QLD86 hap5 against 20WA94 hap26 with over 95% identity when aligned are shown in dark blue. Unique sequences are shown in light blue (hap5) and green (hap26).
(TIFF)

S10 Fig Recombination blocks for USA and Australian haplotypes. Shared haplotype blocks across 14 USA and three Australian haplotypes from *P*. *coronata* f. sp. *avenae* (*Pca*). Shared regions were determined from a threshold of fewer than 50 non-reference variants per 100 kb bin, which were assigned hierarchically from hap1 onto the other 16 haplotypes, then from hap2 onto the next 15 haplotypes, and so on (order: hap1, hap2, hap9, hap10, hap11, hap12, hap13, hap14, hap15, hap16, hap17, hap18, hap19, hap20, hap5, hap6, hap23). Chromosome fill color represents unassigned regions or regions shared with haplotypes earlier in the hierarchy. Percent coverage by preceding haplotypes is shown on the right side. *HD* locus alleles are indicated by the fill color of rectangles at the chromosome 4 midpoint.
(TIFF)

S11 Fig Pangenome saturation estimation using different types of variation. Power Law (red) and Logarithmic (blue) curves fitted to 1000 randomly ordered iterations of saturation analysis for three measures of variation between the 29 unique *P*. *coronata* f. sp. *avenae* (*Pca*) haplotypes: A) gene families, B) haplotype blocks, C) SNPs (for SNPs, hap1 is excluded as it was used as the reference). α is the decay parameter from Heaps Law.
(TIFF)

S12 Fig Redundant unplaced contigs. Examples of three main unplaced contig types: A) extra copies of chromosome 9 repeat-rich region B) extra copies of chromosome 17 ribosomal repeats C) extra copies of other genome sequences which are already represented in chromosomes.
(TIFF)

S1 Methods Protocols for infecting oat with *P*. *coronata* f. sp. *avenae* and infection phenotyping assays. (PDF)

S2 Methods Description of Hi-C spore preparation and sequencing. (PDF)

S3 Methods Procedure for HiFi read decontamination and quality control. (PDF)

S4 Methods Detailed methods for genome assembly and annotation, and manual curation of mating type alleles. (PDF)

S5 Methods Description of pangenome graph variant processing and determination of shared haplotype blocks. (PDF)

S1 Table Isolate metadata. Publication, isolate collection, and sequencing information for 352 *P*. *coronata* f. sp. *avenae* isolates used in this study.
(XLSX)

S2 Table Infection types. Infection type scores for 95 *P*. *coronata* f. sp. *avenae* isolates (rows) on 27 Australian differential oat lines (columns).
(XLSX)

S3 Table Sequencing metadata for genome references. Sequencing metadata for *P*. *coronata* f. sp. *avenae* isolates which were assembled into genome references.
(XLSX)

S4 Table Genome assembly metrics. Length, BUSCO, annotation, and repeat statistics for the 15 *P*. *coronata* f. sp. *avenae* genome references across each of 2 haplotypes and unplaced contigs.
(XLSX)

S5 Table *k*-mer containment results for *STE3*.*2* alleles. *k*-mer containment results for the putative mating type *STE3*.*2* alleles identified in the *P*. *coronata* f. sp. *avenae* genome reference Pca203.
(XLSX)

S6 Table *k*-mer containment results for bWbE locus alleles. *k*-mer containment results for the 13 *bWbE* loci identified in the *P*. *coronata* f. sp. *avenae* haplotype collection. Color gradient reflects spectrum of values between the maximum (red, 100%) and minimum values (white, 90.40% for *k*-mer identity and 12.00% for shared *k*-mers).
(XLSX)

S7 Table Haplotype *k*-mer containment from *Pca* Illumina data. *k*-mer containment results across the 32 available *P*. *coronata* f. sp. *avenae* haplotypes using Illumina data from 352 isolates. Haplotypes were sketched with mash sketch -s 100000. Color gradient reflects spectrum of values between the maximum (red, 100%) and minimum values (white, 99.09% for *k*-mer identity and 82.58% for shared *k*-mers).
(XLSX)

S8 Table Comparison of virulence between lineages L1, L2, and L-TW. Virulence of *P*. *coronata* f. sp. *avenae* isolates from lineages L1, L2, and L-TW proposed to be related through somatic hybridization on the 24 lines in common between the USA differential set and subset of lines included in this study.
(XLSX)

S9 Table Comparison of virulence between lineages L-1990 and L-2017. Virulence of *P*. *coronata* f. sp. *avenae* isolates from lineages L-1990 and L-2017 proposed to be related through somatic hybridization on the USA differential set.
(XLSX)

S10 Table Pairwise shared haplotype sequence. Percent of each *P*. *coronata* f. sp. *avenae* haplotype’s genome sequence (Hap. rows) that is shared by other haplotypes in the haplotype collection (Hap. columns). Shading reflects the range of values (minimum = 0.70 –white; maximum = 55.29 –red). Outlined values are comparisons between haplotypes from the same isolate.
(XLSX)
