# Supplementary figures and images for: A high-resolution haplotype collection uncovers somatic hybridization, recombination and intercontinental movement in oat crown rust
Source: PLoS Genet. 2024 Nov 21;20(11):e1011493. doi: 10.1371/journal.pgen.1011493 (PMC11642970; doi:10.1371/journal.pgen.1011493)

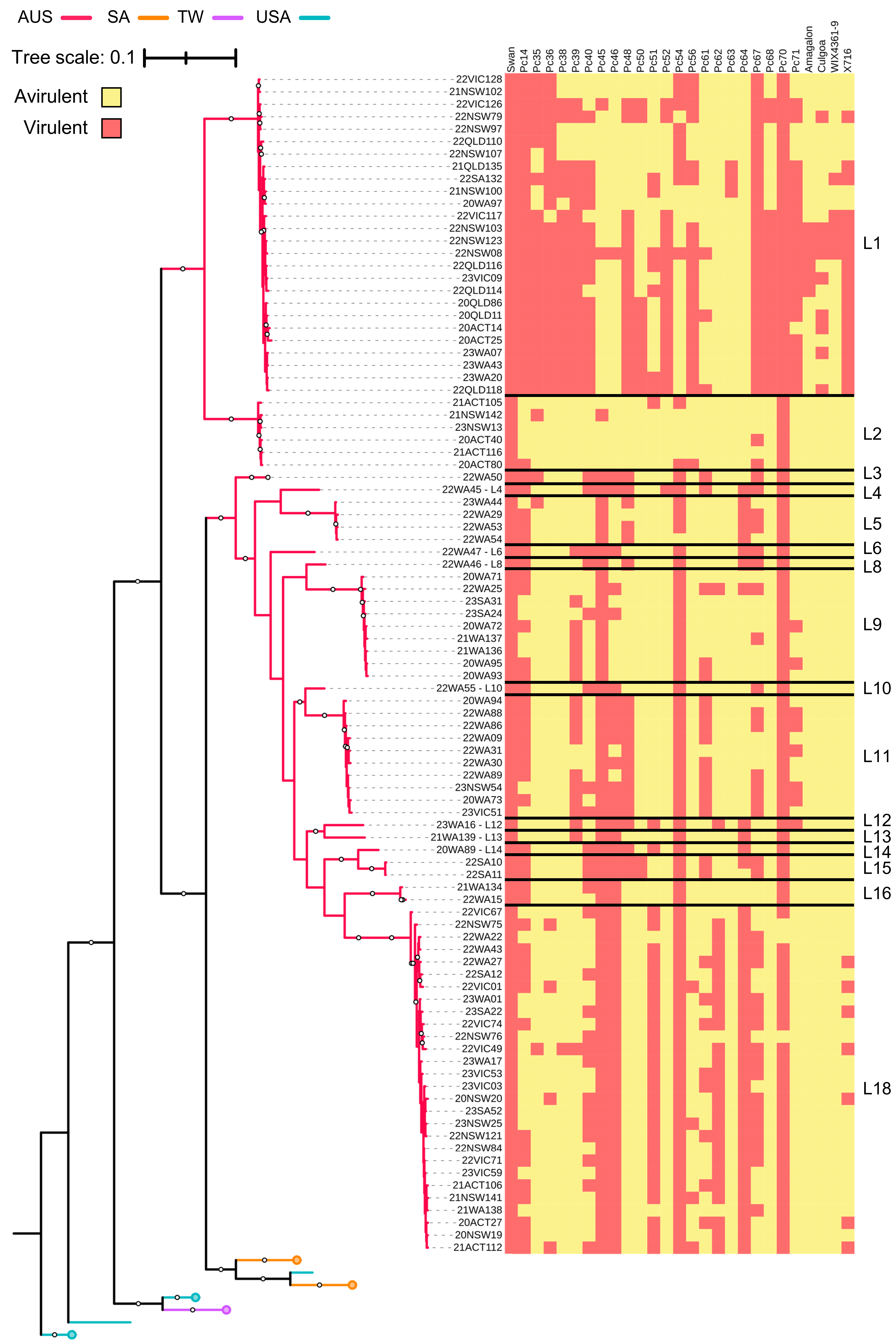

Supplement: S1 Fig — Midpoint rooted Maximum Likelihood phylogenetic tree constructed by mapping reads from 352 P. coronata f. sp. avenae (Pca) isolates and calling variants against the full Pca203 reference (hap1, hap2, and unplaced contigs). 376,646 biallelic SNPs and 500 bootstraps were used. Tree branches are colored by country of origin: AUS = Australia; SA = South Africa; TW = Taiwan; USA = United States of America. Bootstrap values (500 cycles) are percentages (100 = 100%). Tree scale is mean substitutions per site. Heatmap represents isolate virulence on the differential lines (virulent = red, avirulent = yellow). Isolates that were not phenotyped were pruned from the tree, resulting in the removal of L7 and L17. (TIFF) [file pgen.1011493.s001.tiff]

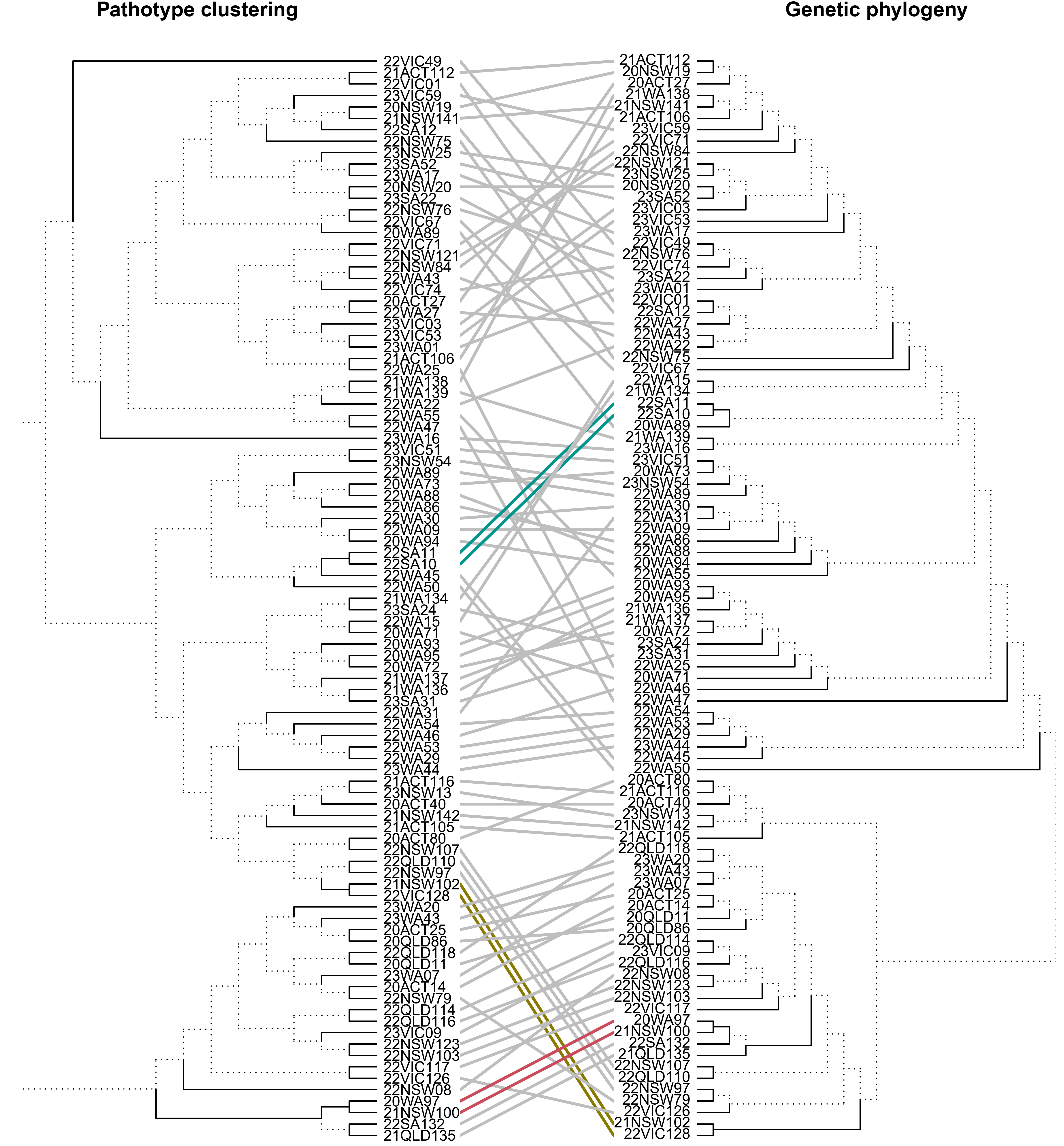

Supplement: S2 Fig — Comparison between tree topologies of 16 Australian P. coronata f. sp. avenae (Pca) lineages from clustering by pathotype (left) versus phylogenetic relationships (right). The Maximum Likelihood tree (right) was generated with 376,646 SNPs from 352 Pca isolates against the complete Pca203 genome (hap1, and hap2, and unplaced contigs), which was pruned to contain only phenotyped Australian isolates and midpoint rooted. The R package ‘tanglegram’ was used to rotate the pathotype clustering tree branches until the best match to the phylogenetic tree was found. Branch lengths are arbitrary in the visualization and assessment of similarity. Solid black lines in the tree structure indicate edges found in both trees. Lines in the center connect the same isolate across trees, with colored lines showing clusters containing more than one isolate which are structurally identical. (TIFF) [file pgen.1011493.s002.tiff]

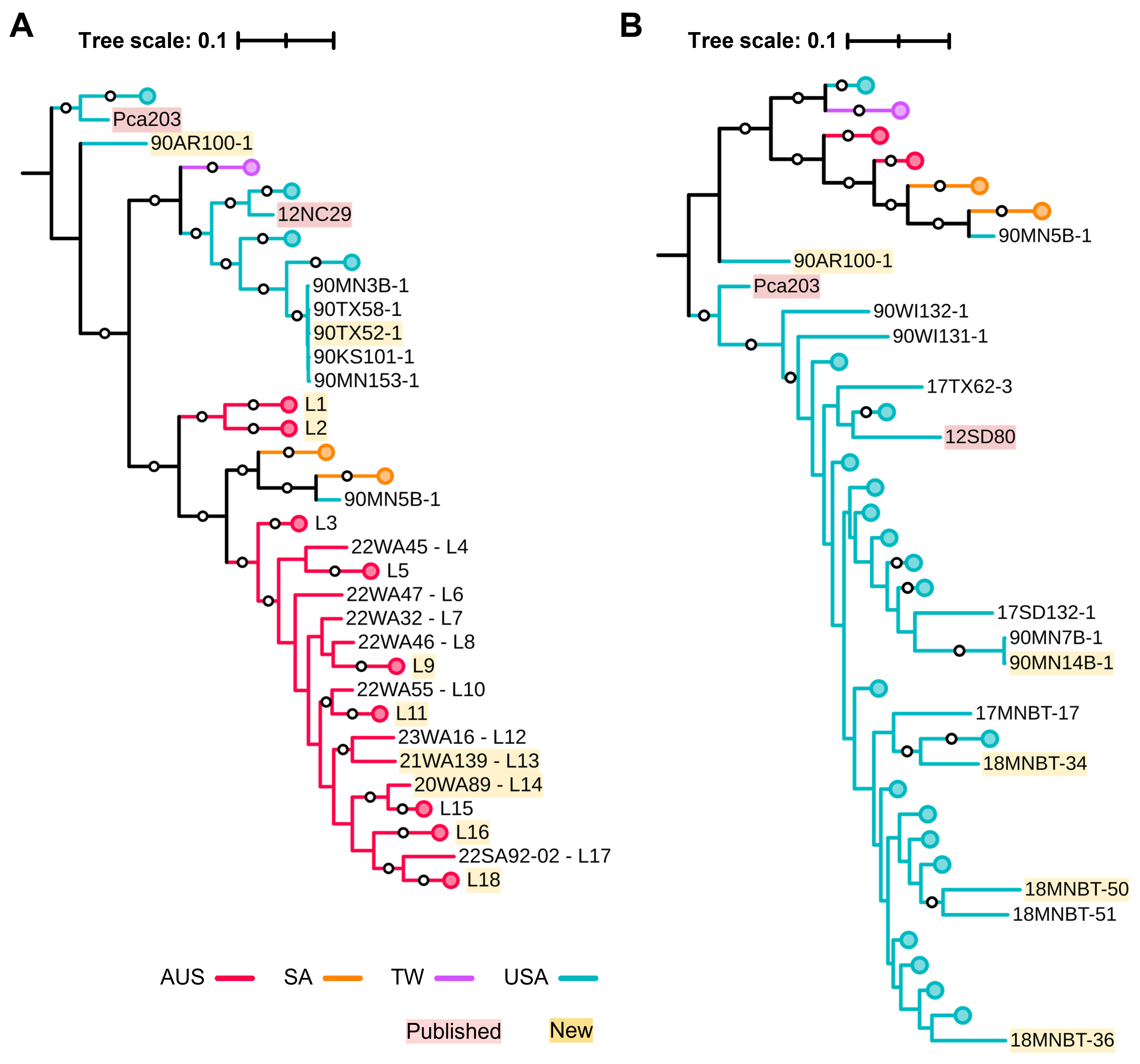

Supplement: S3 Fig — Midpoint rooted Maximum Likelihood phylogenetic tree of 352 P. coronata f. sp. avenae (Pca) isolates constructed by mapping reads and calling variants against the full Pca203 reference (hap1, hap2, and unplaced contigs). 376,646 biallelic SNPs and 500 bootstraps were used. A) Collapsed tree view of primarily Australian lineages (L1 to L18). Except for Pca203, the first two digits of the name of the Pca isolate reflect year of collection, followed by state and sample identifier. B) Collapsed tree view showing USA lineages. USA Pca lineages were not numbered as the population is highly diverse. Tree branches are colored by country of origin: AUS = Australia; SA = South Africa; TW = Taiwan; USA = United States of America. Bootstrap values above 80% shown as white circles. Yellow labels indicate isolates chosen for the haplotype atlas and red labels indicate isolates with published references. Tree scales are mean substitutions per site. (TIFF) [file pgen.1011493.s003.tiff]

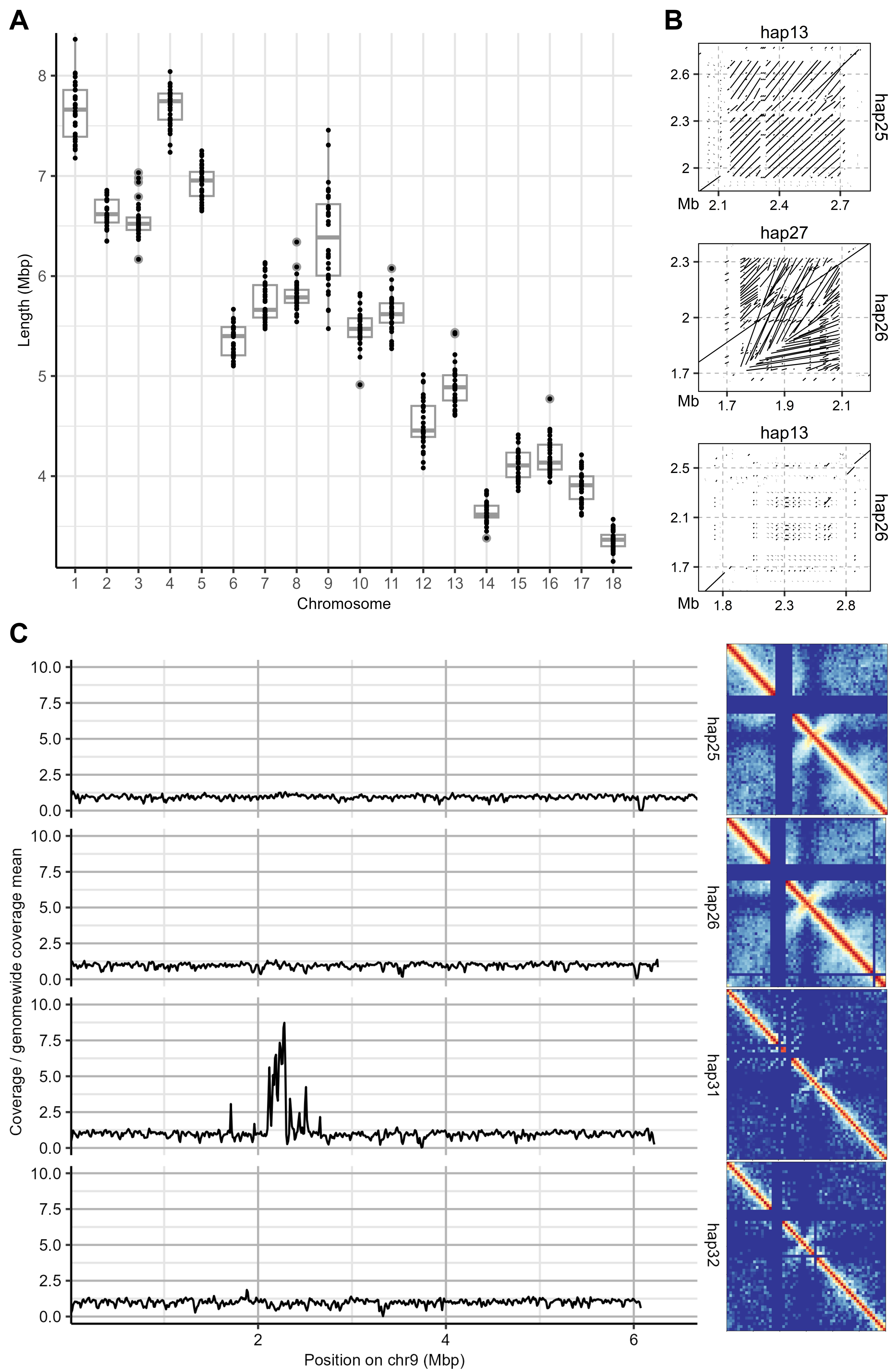

Supplement: S4 Fig — A) Boxplots of chromosome lengths for the 32 P. coronata f. sp. avenae (Pca) haplotypes with black dots indicating chromosome size of each haplotype. Box top and bottom boundaries are the upper and lower quartiles, respectively. Lines within boxes represent the mean. Lines extending below and above boxes delimit minimum and maximum values. B) In descending order: alignments of chromosome 9 regions containing STE3.2 genes between hap13 and hap25 (STE3.2.2), hap26 and hap27 (STE3.2.3), and hap13 and hap26 containing different STE3.2 alleles. C) Ratio between HiFi read coverage within 10 Kb bins and average genome wide coverage on chromosome 9 across four Pca haplotypes (hap25, hap26, hap31, hap32). Chromatin contact maps of chromosome 9 for each haplotype are shown to the right, with colors representing contact frequency (red = high, blue = low). (TIFF) [file pgen.1011493.s004.tiff]

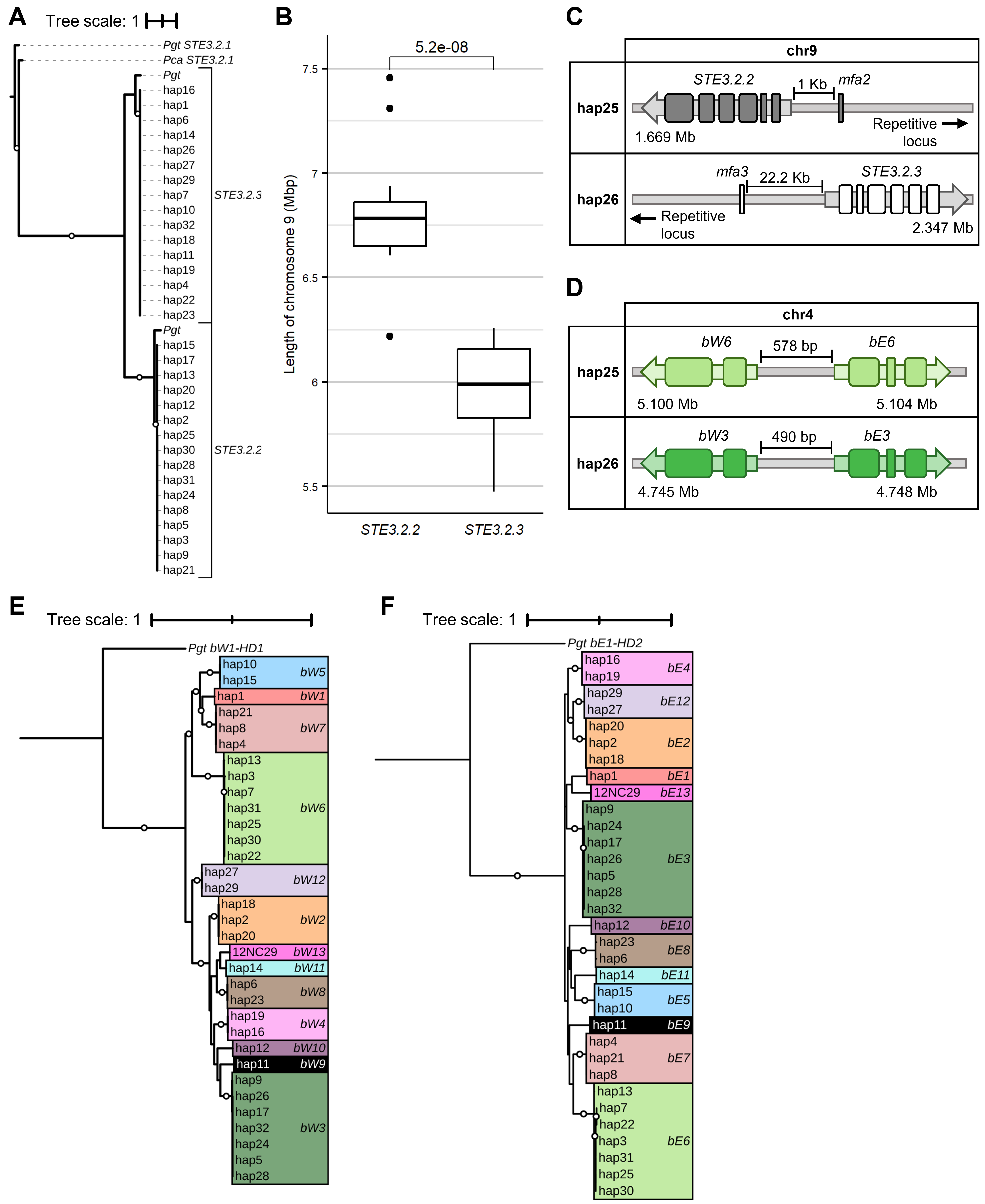

Supplement: S5 Fig — A) Phylogenetic tree of STE3.2 alleles in 32 Pca haplotypes, rooted at P. graminis f. sp. tritici STE3.2.1. B) Boxplots showing chromosome 9 length distribution separated by which STE3.2 allele is present. Box top and bottom boundaries are the upper and lower quartiles, respectively. Lines within boxes represent the mean. Lines extending below and above boxes delimit minimum and maximum values. C) Orientation and arrangement of STE3.2 and mfa (PR) alleles on chromosome 9 (chr9) of hap25 and hap26 from P. coronata f. sp. avenae (Pca) isolate 20WA94. D) Orientation and arrangement of HD alleles on chromosome 4 (chr4) of hap25 and hap26 from Pca isolate 20WA94. E-F) Midpoint-rooted phylogenetic trees of E) bW-HD1 alleles and F) bE-HD2 alleles in 32 Pca haplotypes. Colors indicate identical HD alleles within each tree and allele pairs across trees (i.e. bW3 and bE3 are always found in the same haplotype), as recombination between bW and bE was not detected in our haplotype atlas. Branches with bootstraps (100 cycles) over 80% are shown with white circles at the midpoint. Tree scales are mean substitutions per site. (TIFF) [file pgen.1011493.s005.tiff]

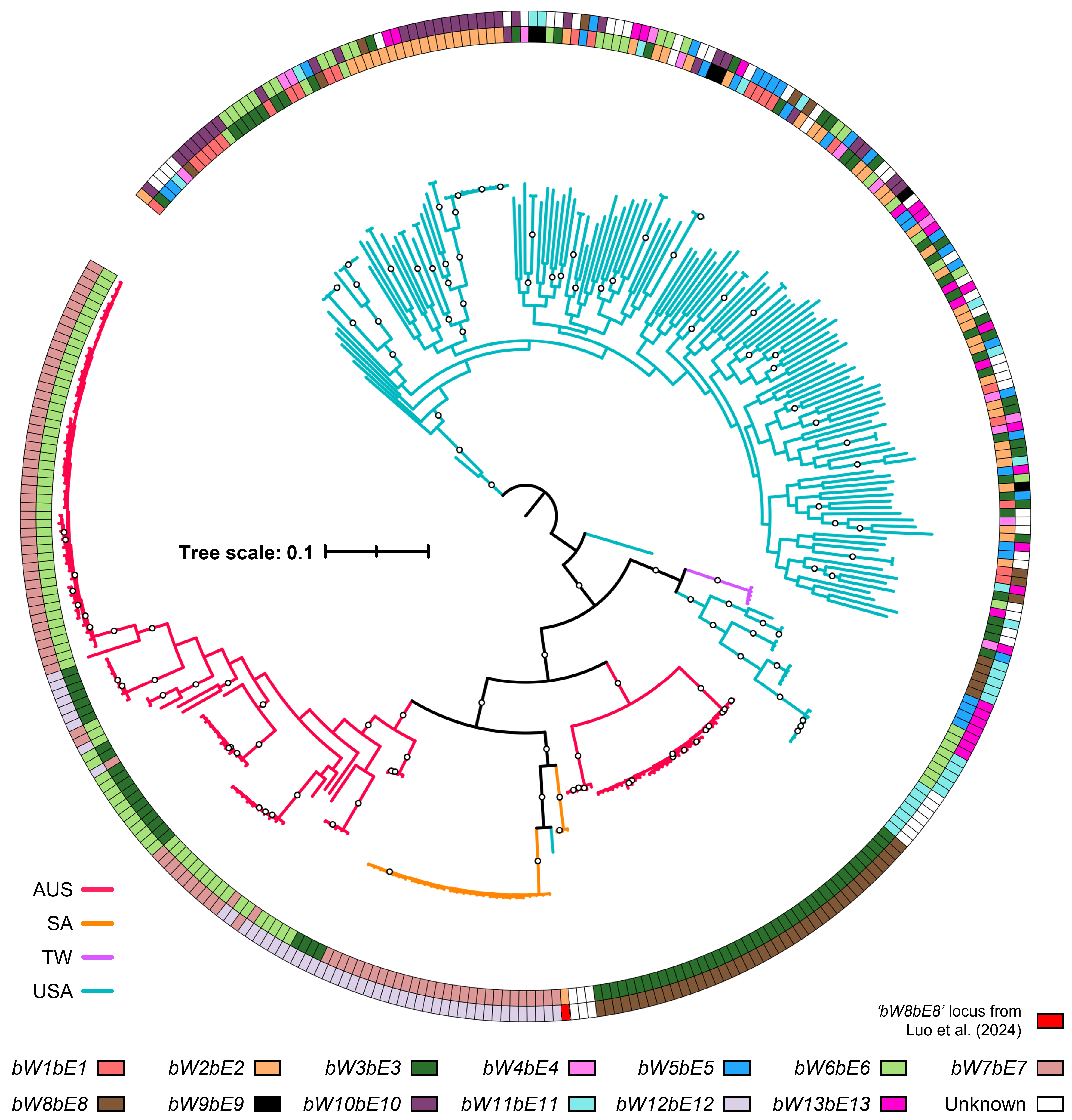

Supplement: S6 Fig — Midpoint rooted phylogenetic tree constructed by mapping short reads from 352 P. coronata f. sp. avenae isolates and calling variants against the complete Pca203 genome (hap1, hap2, unplaced contigs). 376,646 biallelic SNPs were used over 500 bootstraps to produce the Maximum Likelihood tree. Bootstrap values are percentages (100 = 100%). HD alleles are shown as rectangles next to branches. Tree branches are colored by country of origin: AUS = Australia; SA = South Africa; TW = Taiwan; USA = United States of America. Tree scales are mean substitutions per site. (TIFF) [file pgen.1011493.s006.tiff]

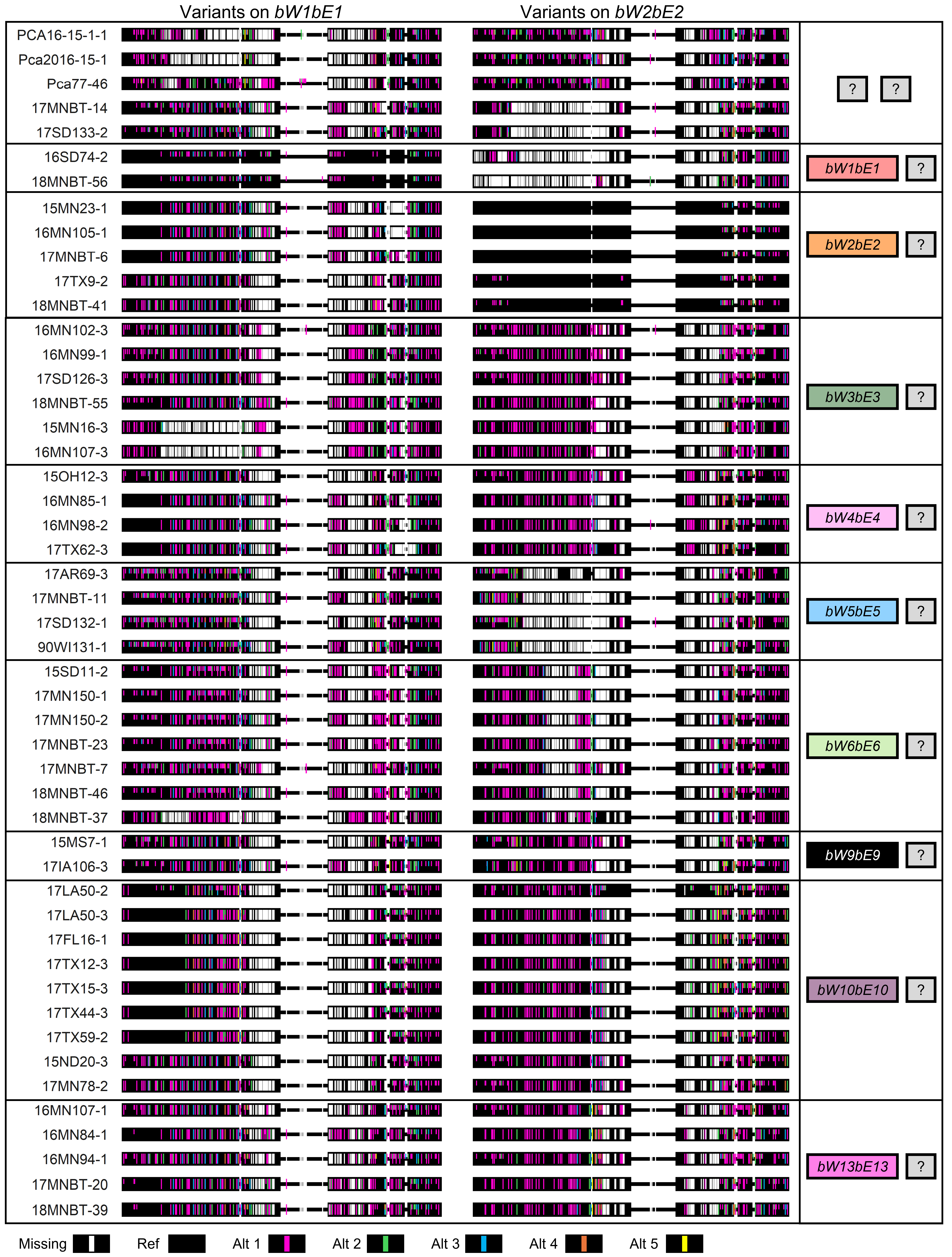

Supplement: S7 Fig — Variants called for 49 P. coronata f. sp. avenae isolates with one or two unknown HD locus alleles in the HD locus regions of Pca203 hap1 (bW1bE1) and hap2 (bW2bE2). Line colors indicate missing (white), reference (black) and alternative (pink, green, blue, orange, yellow) genotypes. Half-length lines indicate heterozygous sites and full-length lines indicate homozygous sites. (TIFF) [file pgen.1011493.s007.tiff]

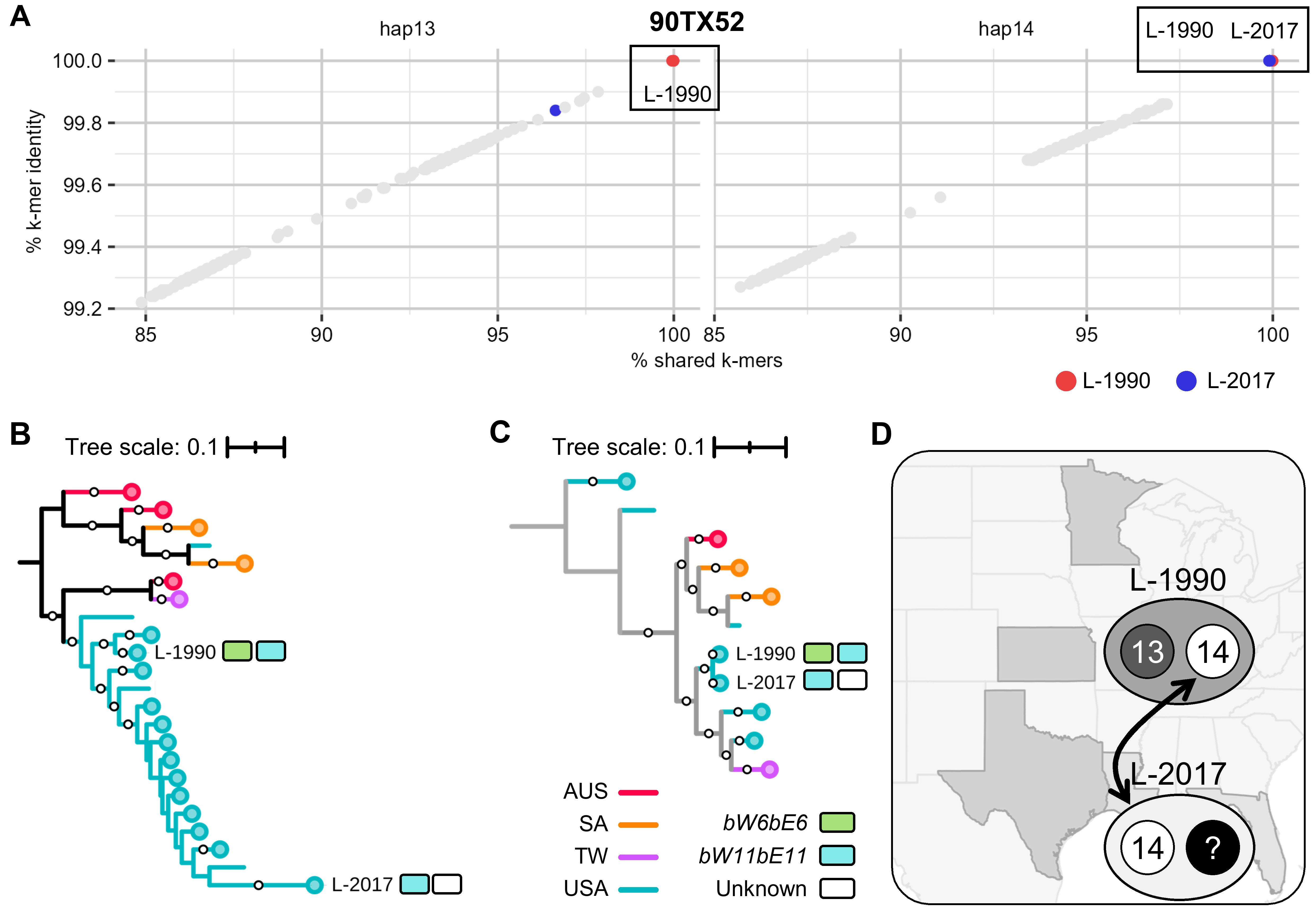

Supplement: S8 Fig — A) Plot of % k-mer identity (y-axis) versus % shared k-mers (x-axis) for short reads from 352 P. coronata f. sp. avenae isolates compared to hap13 and hap14. Colors indicate relevant lineages; light grey points are all other isolates. Midpoint rooted Maximum Likelihood phylogenetic trees constructed from variants from individual haplotypes B) hap13 (194,540 SNPs), C) hap14 (209,542 SNPs) with bootstraps 80% or higher (500 cycles) shown as circles at branch midpoints. Tree branches are colored by country of origin: AUS = Australia; SA = South Africa; TW = Taiwan; USA = United States of America. Collapsed clades are indicated by circles at tips. HD alleles are shown as colored rectangles next to relevant clonal lineages. Tree scales are mean substitutions per site. D) Diagram of the proposed relationship between USA lineages and haplotypes involved in somatic hybridization. (TIFF) [file pgen.1011493.s008.tiff]

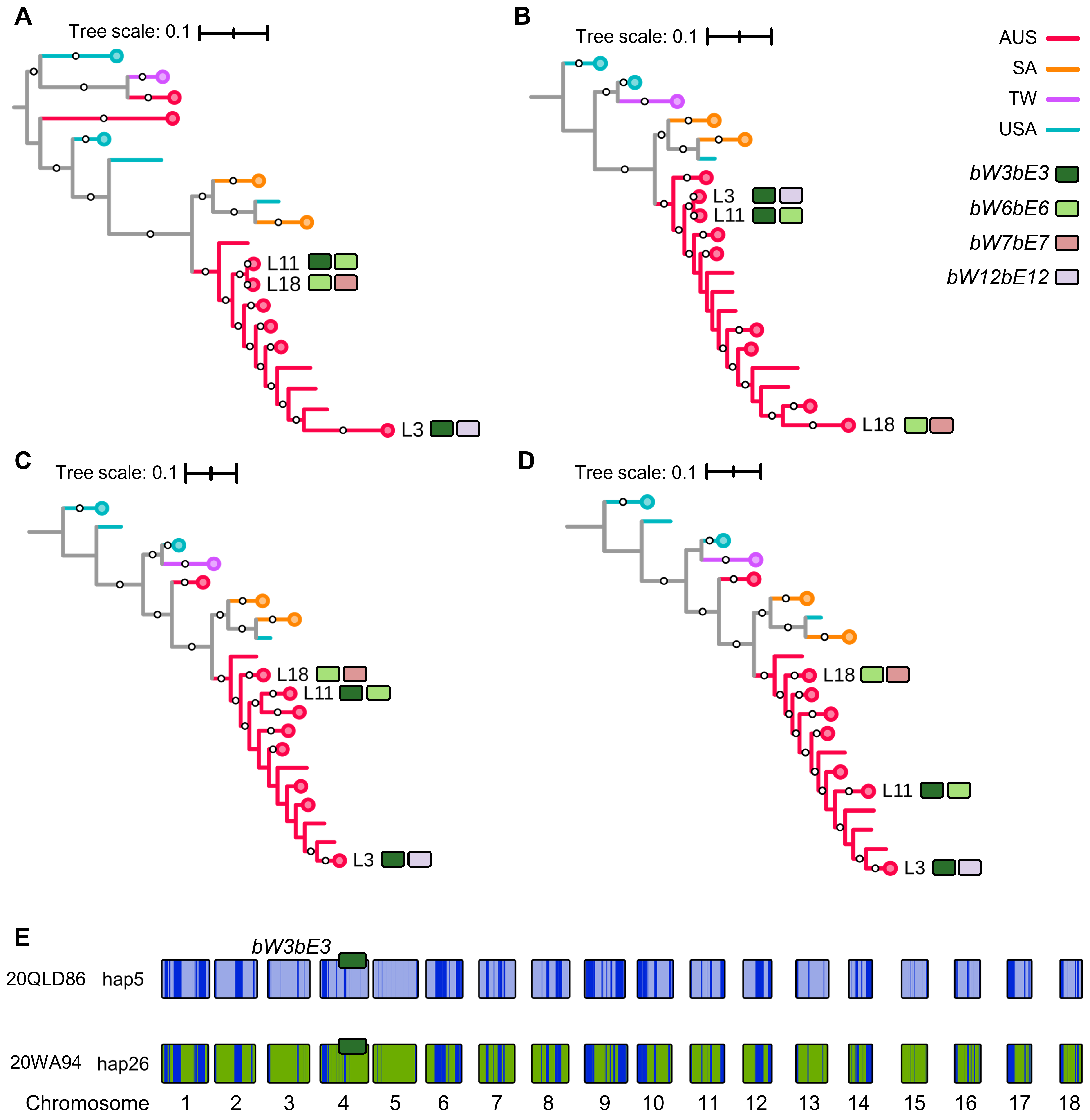

Supplement: S9 Fig — Midpoint rooted Maximum Likelihood phylogenetic trees for 352 P. coronata f. sp. avenae isolates constructed from variants from individual haplotypes A) hap25 (205,993 SNPs) B) hap26 (188,803 SNPs) C) hap3 (228,300 SNPs) D) hap4 (226,617 SNPs). Bootstrap supports 80% or higher (500 cycles) are shown as circles at branch midpoints. Tree branches are colored by country of origin: AUS = Australia; SA = South Africa; TW = Taiwan; USA = United States of America. Collapsed branches are indicated by circles at tips. HD alleles are shown as colored rectangles next to relevant clonal lineages. Tree scales are mean substitutions per site. E) Regions of 20QLD86 hap5 against 20WA94 hap26 with over 95% identity when aligned are shown in dark blue. Unique sequences are shown in light blue (hap5) and green (hap26). (TIFF) [file pgen.1011493.s009.tiff]

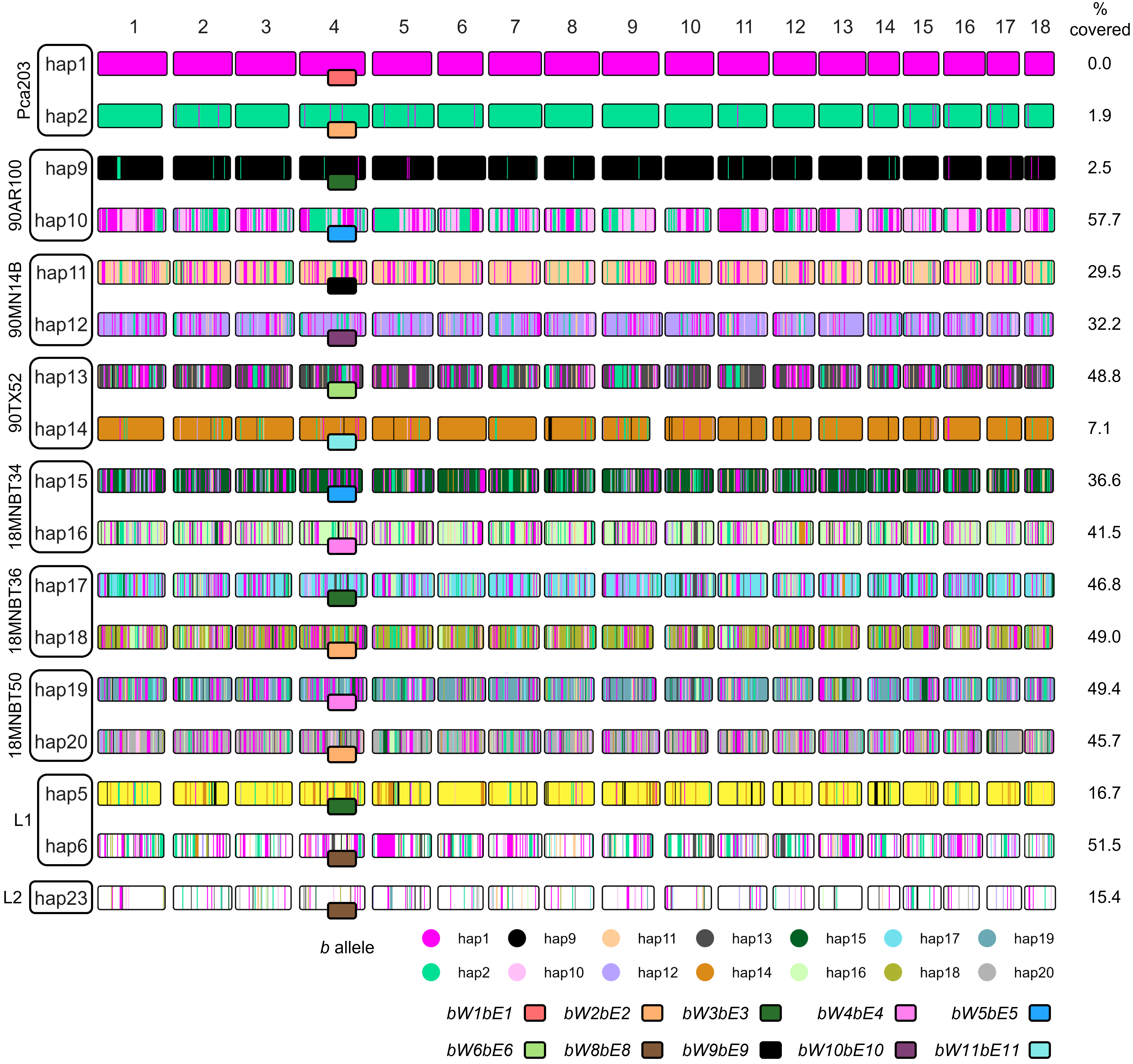

Supplement: S10 Fig — Shared haplotype blocks across 14 USA and three Australian haplotypes from P. coronata f. sp. avenae (Pca). Shared regions were determined from a threshold of fewer than 50 non-reference variants per 100 kb bin, which were assigned hierarchically from hap1 onto the other 16 haplotypes, then from hap2 onto the next 15 haplotypes, and so on (order: hap1, hap2, hap9, hap10, hap11, hap12, hap13, hap14, hap15, hap16, hap17, hap18, hap19, hap20, hap5, hap6, hap23). Chromosome fill color represents unassigned regions or regions shared with haplotypes earlier in the hierarchy. Percent coverage by preceding haplotypes is shown on the right side. HD locus alleles are indicated by the fill color of rectangles at the chromosome 4 midpoint. (TIFF) [file pgen.1011493.s010.tiff]

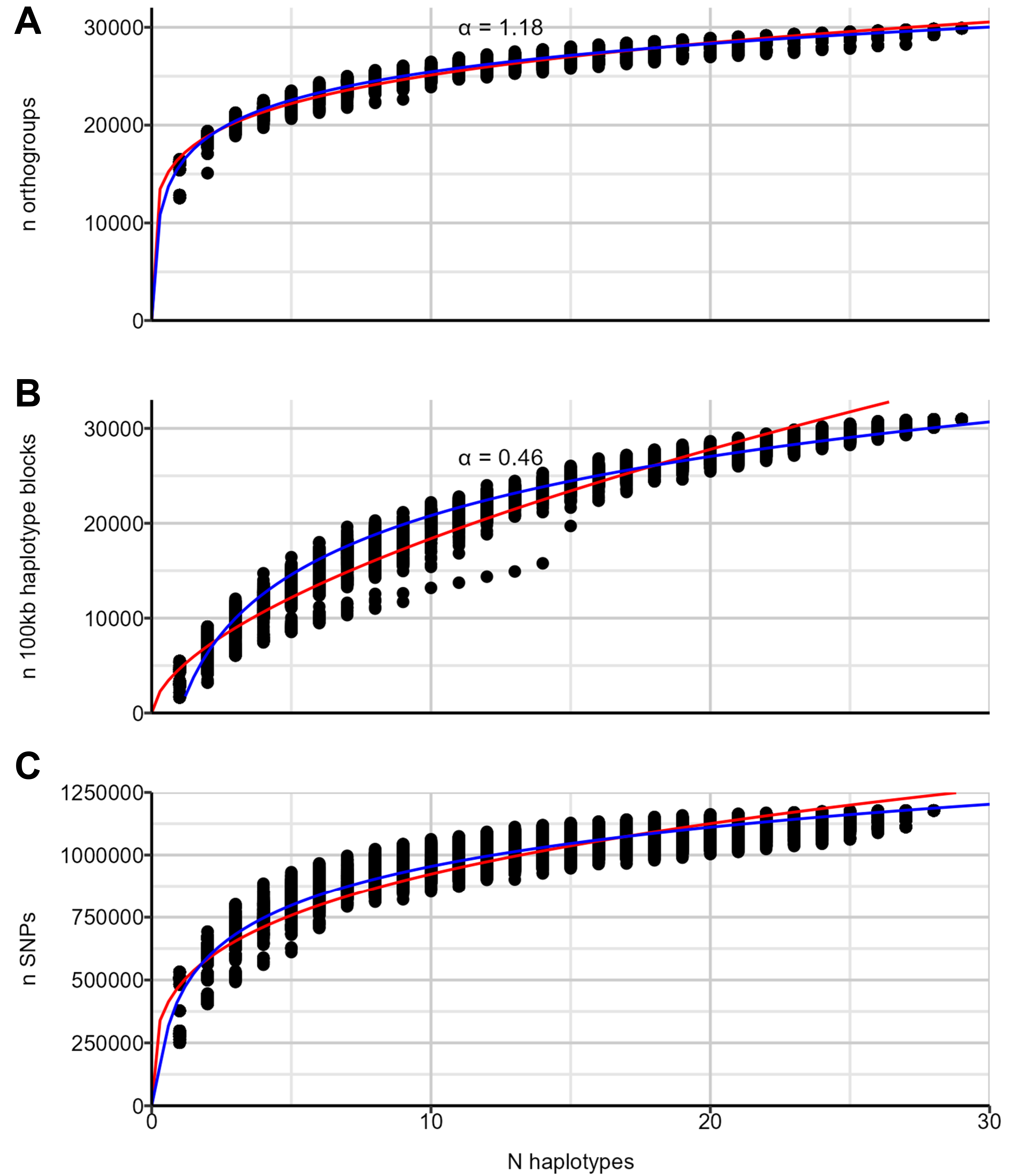

Supplement: S11 Fig — Power Law (red) and Logarithmic (blue) curves fitted to 1000 randomly ordered iterations of saturation analysis for three measures of variation between the 29 unique P. coronata f. sp. avenae (Pca) haplotypes: A) gene families, B) haplotype blocks, C) SNPs (for SNPs, hap1 is excluded as it was used as the reference). α is the decay parameter from Heaps Law. (TIFF) [file pgen.1011493.s011.tiff]

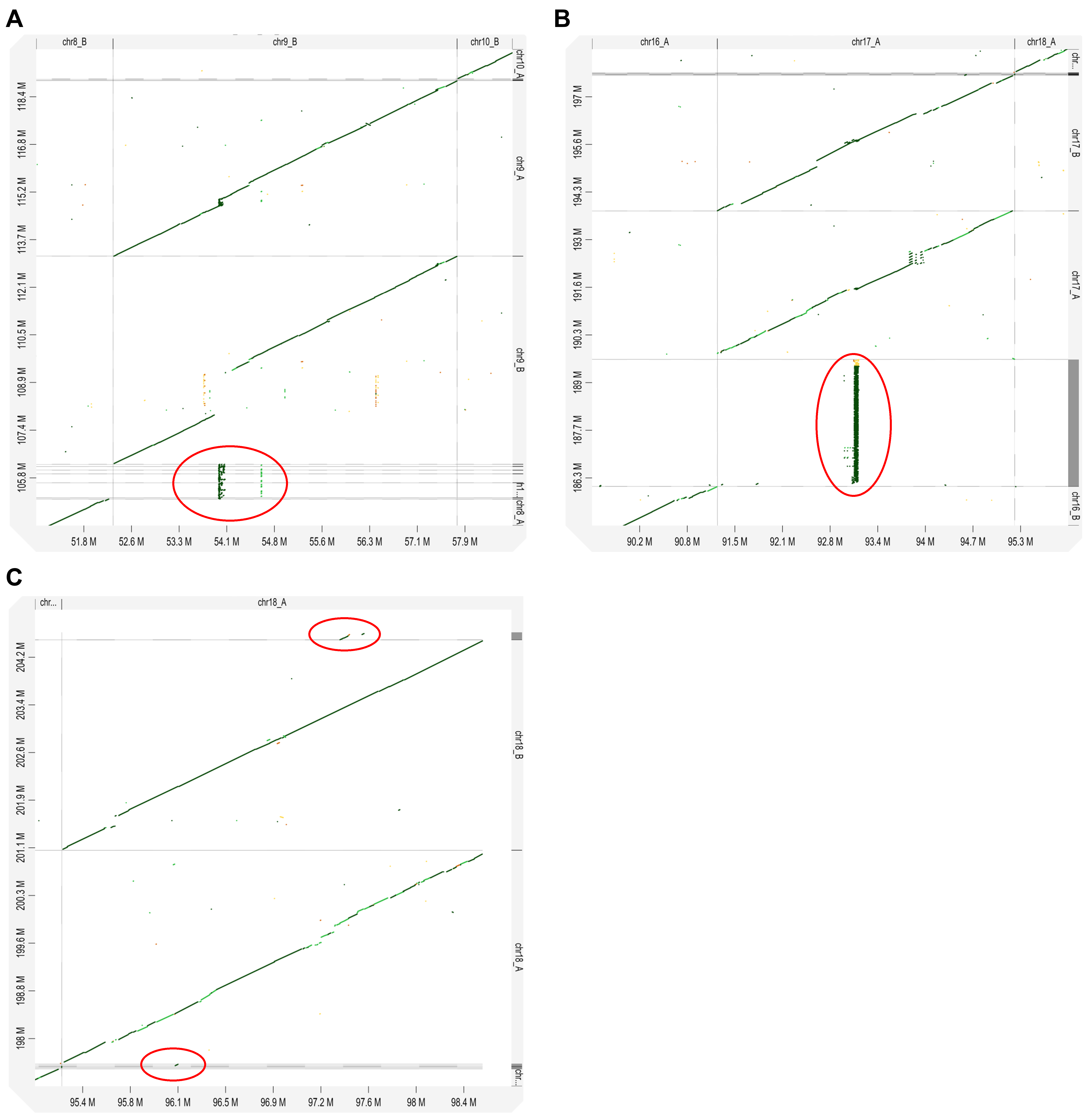

Supplement: S12 Fig — Examples of three main unplaced contig types: A) extra copies of chromosome 9 repeat-rich region B) extra copies of chromosome 17 ribosomal repeats C) extra copies of other genome sequences which are already represented in chromosomes. (TIFF) [file pgen.1011493.s012.tiff]
