# Supplementary material for: A high-resolution haplotype collection uncovers somatic hybridization, recombination and intercontinental movement in oat crown rust
Source: PLoS Genet. 2024 Nov 21;20(11):e1011493. doi: 10.1371/journal.pgen.1011493 (PMC11642970; doi:10.1371/journal.pgen.1011493)
Supplement: S1 Methods — (PDF) [file pgen.1011493.s013.pdf]

S1 Methods

Plant growth conditions and phenotyping assays

Susceptible oat cultivars that were used for amplification vary across countries due to differences in environmental conditions that affect host susceptibility. ‘Marvelous’ was used in the USA, ‘Swan’ in Australia, and ‘NTU Selection 1’ and ‘Swan’ were used in Taiwan. For revival of field samples, the submitted infected leaves were cut into fragments and combined with 1-2 mL Novec oil. The oil mixture was pipetted onto susceptible oat genotypes and the plants were air dried for 30 minutes, misted with water, and kept in humidity chambers (90-99% humidity) for two days before removal to growth cabinets at 23C° for 16 hours light and 18C° for 8 hours dark. After nine days, rust was collected. To isolate single pustules from bulked samples, plants were treated with 15 mL maleic hydrazide per pot and a dilution of 0.5 mg spores in 600 uL of Novec oil was pipetted onto susceptible plants at 9 days of growth. Misting and humidity chamber treatments were the same. Plants were trimmed at around seven days post inoculation (dpi) to keep only leaves with sparse single pustules. Wax paper was scraped against the pustule and leaf surface for collection; these isolations were immediately used to infect new plants. Subsequent infections were performed with approximately 20 mg spores in 400 uL of Novec oil with the same conditions as described before.

For phenotyping assays, 5-6 seeds per genotype were planted with four genotypes per pot. At 9 days post inoculated the plants were treated with 15 mL maleic hydrazide per pot and misted with water, and then a mixture of 50 mg spores with 150 mg talcum powder was spread onto the plants through Miracloth (Millipore Calbiochem®). At 10-11 dpi, infection types were recorded. Multiple infection types may be observed on a

single leaf or across biological replicates of the same genotype. As long as infection types do not segregate across the resistant/susceptible boundary, the most common infection type is taken. For example, if four of five leaves have primarily fleck (;) infection types and one leaf has minor sporulation (1), the infection type recorded is fleck. If infection types segregated between resistance and susceptibility across biological replicates (i.e. three plants with score of ‘4’, two plants with score of ‘1’), we repeated the phenotyping for that genotype to ensure an accurate score.
