# Supplementary material for: A high-resolution haplotype collection uncovers somatic hybridization, recombination and intercontinental movement in oat crown rust
Source: PLoS Genet. 2024 Nov 21;20(11):e1011493. doi: 10.1371/journal.pgen.1011493 (PMC11642970; doi:10.1371/journal.pgen.1011493)
Supplement: S2 Methods — (PDF) [file pgen.1011493.s014.pdf]

S2 Methods

Sample and library preparation for Hi-C sequencing

Spores for Hi-C were prepared by suspending 150-200 mg of urediniospores in 13 mL 1% formaldehyde, which was incubated for 20 minutes with periodic vortexing. Glycine powder was added to 1g/100 mL concentrated to quench crosslinking with a 15 minute incubation, again with periodic vortexing. The tubes were spun at 1000 g for two minutes and as much liquid as possible was removed taking care to avoid the clumps of rust spores, which are hydrophobic and tend to float to the top of solutions. The spores were rinsed with 10 mL of water and spun as before. As much water as possible was removed by pipetting. Treated spores were ground with a chilled mortar and pestle with liquid nitrogen added periodically to maintain cold temperature. Samples were kept at -80C° and shipped on dry ice to library preparation providers. Hi-C libraries for isolates 18MNBT34, 18MNBT36, 18MNBT50, 90AR100, 90MN4b, 90TX52, 20NSW19, 20QLD86, and 20WA72 were prepared at Phase Genomics, Seattle WA USA and sequenced with Illumina Novaseq by Azenta Life Sciences (formerly Genewiz). Libraries for 20WA94, 21ACT116, and 21WA134 were prepared and sequenced with Illumina Novaseq at the Ramaciotti Centre for Genomics, NSW Australia. Hi-C for 20WA95 was not prepared, as it is a clone of 20WA72. During the genome assembly pipeline, Hi-C data from 20WA72 was used with 20WA95 HiFi data.
