# Supplementary material for: A high-resolution haplotype collection uncovers somatic hybridization, recombination and intercontinental movement in oat crown rust
Source: PLoS Genet. 2024 Nov 21;20(11):e1011493. doi: 10.1371/journal.pgen.1011493 (PMC11642970; doi:10.1371/journal.pgen.1011493)
Supplement: S3 Methods — (PDF) [file pgen.1011493.s015.pdf]

S3 Methods

Filtering PacBio HiFi reads with short reads using *k*-mer containment

Initial genome assemblies of 20WA89, 21ACT116, and 21WA134 were larger than expected and showed more fragmentation than assemblies for other isolates performed with the same procedures. To identify potential contaminant isolates, extra contigs (i.e. third copy of segments from main chromosome sequences) were screened with mash v2.0 [1] against short-read data for individuals from different lineages. Those with the highest containment across the greatest number of extra contigs were chosen for use in filtering HiFi reads. Each HiFi read was sketched individually (-s 1000) with mash (v2.0) screen and Illumina reads for the suspected contaminants were screened against the HiFi reads [1]. *k*-mer identity cutoffs were then applied and tested iteratively by creating a draft assembly with the cleaned reads to assess the success of removal, and the parameters resulting in the best test assembly were chosen to generate the filtered read set. Filtering was deemed adequate when diploid assembly size and contiguity with the filtered reads was comparable to that of pure samples. Percent shared *k*-mers were not considered in filtering as the sequences represented by HiFi and short read sequencing are not expected to overlap perfectly. Final HiFi read filtering parameters were as follows:

- 20WA89 HiFi reads were screened with 20WA89, 22WA47, and 22WA32 Illumina data and were retained if 20WA89 *k*-mer identity was highest or > 0.9998.
- 21ACT116 HiFi reads were screened with 21ACT116, 21WA134, and 20WA94 Illumina data and were retained if 21ACT116 *k*-mer identity was highest or > 0.9999.

- 21WA134 HiFi reads were screened with 21WA134, 22WA15, and 20WA72 and were retained if 21WA134 or 22WA15 *k*-mer identity was highest or > 0.9999.

Approximately 21% of 20WA89 HiFi reads longer than 10 Kb were removed with the remaining reads randomly downsampled to 40X coverage. For 21WA134, 11% of the reads longer than 10 Kb were removed and remaining reads were downsampled to 30X coverage. Finally, 38% of 21ACT116 reads longer than 10 Kb were removed and downsampled to around 30X coverage. Downsampling was performed with seqkit v2.7.0 [2].

References

1. Ondov BD, Starrett GJ, Sappington A, Kostic A, Koren S, Buck CB, et al. Mash Screen: high-throughput sequence containment estimation for genome discovery. Genome Biol. 2019;20: 232. doi:10.1186/s13059-019-1841-x

2. Shen W, Le S, Li Y, Hu F. SeqKit: a cross-platform and ultrafast toolkit for FASTA/Q file manipulation. PLoS One. 2016;11: e0163962. doi:10.1371/journal.pone.0163962
