# Supplementary material for: A high-resolution haplotype collection uncovers somatic hybridization, recombination and intercontinental movement in oat crown rust
Source: PLoS Genet. 2024 Nov 21;20(11):e1011493. doi: 10.1371/journal.pgen.1011493 (PMC11642970; doi:10.1371/journal.pgen.1011493)
Supplement: S4 Methods — (PDF) [file pgen.1011493.s016.pdf]

S4 Methods

Genome assembly, filtering contigs, nuclear phasing, and scaffolding

PacBio HiFi reads for most isolates were input into hifiasm v0.16.1 [1] with their respective Hi-C reads integrated for all samples except 20WA95, for which the Hi-C data from its clonal relative 20WA72 was used. For 20WA89, 20WA94, 21ACT116, 21WA134, and 21WA139, the same was performed with hifiasm v0.19.5 [1]. Haplotypes resulting from hifiasm assembly for each isolate were combined and contigs belonging to the mitochondrial genome were filtered using BLAST+ v2.13.0 [2]. The PacBio reads were mapped back to the assembly using minimap2 v2.22 (--ax --secondary=off) [3] and contig coverage was calculated with bbmap (v39.06) pileup (sourceforge.net/projects/bbmap/). Contigs with average coverage of <= 5X were removed, except 20WA89, 20WA94, 21ACT116, and 21WA134, which used a cutoff of <= 10X. The remaining contigs were then BLASTed to the NCBI nucleotide database with blast+ v2.13.0 and contigs without ‘Puccinia’, ‘Medioppia’, ‘Phakopsora’, ‘Melampsora’, ‘Uromyces’, or ‘ribosomal’ in their top five hits were removed [2,4]. The cleaned assembly was prepared and phased with NuclearPhaser v1.1 (https://github.com/JanaSperschneider/NuclearPhaser). Scaffolding was completed by aligning each haplotype to the Pca203 ‘A’ genome (hap1) with D Genies v1.5.0 to determine contig order and orientation, in addition to generating Hi-C contact maps with HiC-Pro v3.1.0 and hicexplorer v3.7.2 [5–7]. Unplaced contigs were excluded from the analysis of haplotypes as they were already represented in the chromosomes, were on average short (8,024- 633,353 bp, mean = 43.83 Kb) and had high repeat content (55.60-91.16%, mean = 81.08%) (S1 Fig and S4 Table).

Repeat masking and genome annotation

Repeats in the scaffolded genomes were masked with repeatmodeler v2.0.2a and repeatmasker v4.1.2pl (--nolow), retaining only classified repeats [8,9]. RNAseq reads from 12NC29 (spores, haustoria) and Pca203 (5 dpi) were used [10,11]. RNAseq reads from 12NC29 and Pca203 were mapped to individual haplotypes and unplaced contigs separately with Hisat2 v2.2.1 (--max-intronlen 3000 --dta --no-unal --rna-strandedness RF) [10–12]. Read mappings were merged and used as input for Trinity v2.13.2 in genome-guided mode (--jaccard_clip --genome_guided_max_intron 3000 --SS_lib_type RF) [13]. Hisat2 v2.2.1 (--max-intronlen 3000 --dta --no-una) was also used to align the RNAseq reads in preparation for assembly with stringtie v2.2.1 (-s 1 -m 150) [12,14]. Codingquarry v2.0 was run on infection and spore transcripts separately [15] and filtered as described in [16]. Funannotate v1.8.5 training was run on previous Trinity transcripts (--stranded RF --no_trimmomatic --jaccard_clip) [17]. Funannotate v1.8.5 predict was run using on the deduplicated and repeatmasked genomes with the Trinity, coding quarry, and pucciniomycotina EST evidence (--ploidy 2 --optimize_augustus --busco_seed_species ustilago --weights pasa:10 codingquarry:0). Funnanotate v1.8.13 update was run with this training information. Finally, annotations were finalized by processing with transdecoder v5.5.0 and agat v1.0.0 (Haas, BJ https://github.com/TransDecoder/TransDecoder) [18].

Manual annotation of mating type alleles

Pca203-*STE3.2.2* was not annotated correctly due to an overlapping gene annotated on the opposite strand; the correct gene model for *STE3.2.2* was recovered from augustus predictions and confirmed by alignment to *Puccinia graminis* f. sp. *tritici*

alleles with CLUSTALW [19]. *STE3.2.2* required recovery and/or correction in nearly all isolates and some haplotypes had incorrect exon boundaries in *STE3.2.3* which were corrected. The 5' ends of *bW-HD1* and *bE-HD2* are highly variable and because RNAseq data from 12NC29 and Pca203 were used for annotation of all haplotypes, several gene models were recovered from augustus, coding quarry, or pasa predictions and/or manually corrected using conserved domain information and protein alignments. Preliminary screens indicated that one *HD* allele pair for the published reference 12NC29 [10] was not sampled in any of the chromosome-level references; as such, the missing allele was identified in the 12NC29 contig-level reference genome, and the locus was added to the screening.

References

1. Cheng H, Concepcion GT, Feng X, Zhang H, Li H. Haplotype-resolved *de novo* assembly using phased assembly graphs with hifiasm. Nat Methods. 2021;18: 170-175. doi:10.1038/s41592-020-01056-5

2. Camacho C, Coulouris G, Avagyan V, Ma N, Papadopoulos J, Bealer K, et al. BLAST+: architecture and applications. BMC Bioinformatics. 2009;10: 421. doi:10.1186/1471-2105-10-421

3. Li H. Minimap2: pairwise alignment for nucleotide sequences. Bioinformatics. 2018;34: 3094–3100. doi:10.1093/bioinformatics/bty191

4. Sayers EW, Bolton EE, Brister JR, Canese K, Chan J, Comeau DC, et al. Database resources of the national center for biotechnology information. Nucleic Acids Res. 2022;50: D20–D26. doi:10.1093/nar/gkab1112

5. Servant N, Varoquaux N, Lajoie BR, Viara E, Chen C-J, Vert J-P, et al. HiC-Pro: an optimized and flexible pipeline for Hi-C data processing. Genome Biol. 2015;16:259. doi:10.1186/s13059-015-0831-x

6. Cabanettes F, Klopp C. D-GENIES: dot plot large genomes in an interactive, efficient and simple way. PeerJ. 2018;6: e4958. doi:10.7717/peerj.4958

7. Ramírez F, Bhardwaj V, Arrigoni L, Lam KC, Grüning BA, Villaveces J, et al. High-resolution TADs reveal DNA sequences underlying genome organization in flies. Nat Commun. 2018;9: 189. doi:10.1038/s41467-017-02525-w

8. Smit A, Hubley R, Green P. Repeatmasker Open-4.0 v4.1.2-pl. 2013 [cited 29 Apr 2024]. Available: https://www.repeatmasker.org/

9. Flynn JM, Hubley R, Goubert C, Rosen J, Clark AG, Feschotte C, et al. RepeatModeler2 for automated genomic discovery of transposable element families. Proceedings of the National Academy of Sciences. 2020;117: 9451–9457. doi:10.1073/pnas.1921046117

10. Miller ME, Ying Z, Vahid O, Jana S, Benjamin S, Castle R, et al. *De novo* assembly and phasing of dikaryotic genomes from two isolates of *Puccinia coronata* f. sp. *avenae*, the causal agent of oat crown rust. mBio. 2018;9. doi:10.1128/mbio.01650-17

11. Henningsen EC, Hewitt T, Dugyala S, Nazareno ES, Gilbert E, Li F, et al. A chromosome-level, fully phased genome assembly of the oat crown rust fungus *Puccinia coronata* f. sp. *avenae*: a resource to enable comparative genomics in the cereal rusts. G3 Genes|Genomes|Genetics. 2022;12: jkac149. doi:10.1093/g3journal/jkac149

12. Kim D, Paggi JM, Park C, Bennett C, Salzberg SL. Graph-based genome alignment and genotyping with HISAT2 and HISAT-genotype. Nat Biotechnol. 2019;37: 907–915. doi:10.1038/s41587-019-0201-4

13. Grabherr MG, Haas BJ, Yassour M, Levin JZ, Thompson DA, Amit I, et al. Full-length transcriptome assembly from RNA-Seq data without a reference genome. Nat Biotechnol. 2011;29: 644–652. doi:10.1038/nbt.1883

14. Pertea M, Pertea GM, Antonescu CM, Chang T-C, Mendell JT, Salzberg SL. StringTie enables improved reconstruction of a transcriptome from RNA-seq reads. Nat Biotechnol. 2015;33: 290–295. doi:10.1038/nbt.3122

15. Testa AC, Hane JK, Ellwood SR, Oliver RP. CodingQuarry: highly accurate hidden Markov model gene prediction in fungal genomes using RNA-seq transcripts. BMC Genomics. 2015;16: 170. doi:10.1186/s12864-015-1344-4

16. Sperschneider J, Hewitt T, Lewis DC, Periyannan S, Milgate AW, Hickey LT, et al. Nuclear exchange generates population diversity in the wheat leaf rust pathogen *Puccinia triticina*. Nat Microbiol. 2023;8: 2130–2141. doi:10.1038/s41564-023-01494-9

17. Palmer JM, Stajich J. Funannotate v1.8.1: eukaryotic genome annotation. Zenodo; 2020. Available: https://doi.org/10.5281/zenodo.4054262

18. Dainat J, Hereñú D, Davis E, Crouch K, Sol L, Agostinho N, et al. NBISweden/AGAT: AGAT-v1.1.0. Zenodo; 2023. doi:10.5281/zenodo.7950165

19. Larkin MA, Blackshields G, Brown NP, Chenna R, McGettigan PA, McWilliam H, et al. Clustal W and Clustal X version 2.0. Bioinformatics. 2007;23: 2947–2948. doi:10.1093/bioinformatics/btm404
