# Supplementary material for: A high-resolution haplotype collection uncovers somatic hybridization, recombination and intercontinental movement in oat crown rust
Source: PLoS Genet. 2024 Nov 21;20(11):e1011493. doi: 10.1371/journal.pgen.1011493 (PMC11642970; doi:10.1371/journal.pgen.1011493)
Supplement: S5 Methods — (PDF) [file pgen.1011493.s017.pdf]

S5 Methods

Pangenome graph approach for variant calling and recombination block analysis

The cactus-pangenome pipeline generates a pangenome graph and can also call variants with each input genome as the “reference”, using the other genomes as “samples”. By listing every haplotype as a “reference”, 32 VCF files were generated that map variants to physical positions along each haplotype. Variants along each reference were binned every 100 Kb and bins with <= 50 non-reference SNPs were marked as “shared” between the reference and the sample haplotype. This threshold was determined by visualizing non-reference SNPs distributions across hap25, where hap3 and hap4 are samples and hap25 is the reference. Histogram bins without any SNP calls were ignored to prevent repetitive or poorly aligned regions from being assigned as shared. Adjacent bins that were marked as shared for each sample-reference pair were combined with bedtools (v2.31.1) merge [1]. The intermediate files produced from this are pairwise shared haplotype blocks between all haplotypes. However, to produce a sensible visualization, a hierarchy must be applied to handle overlaps between shared haplotype blocks. For example, hypothetical haplotypes hapA and hapB have a region in common. HapC also shares part of this region and additionally shares sequence adjacent to this with hapB. In a hierarchy, we could place hapA at the top, so the sequence shared by all three haplotypes is colored as “hapA” and only the non-overlapping section shared between hapC and hapB is colored as “hapB”. The order of the hierarchy we used was determined by calculating the proportion of each haplotype covered by others without removing overlaps, which is shown in S10 Table. Using a custom script, these regions were then subjected to an

ordered bedtools subtraction, whereby regions from the first haplotype in the hierarchy are assigned first across all other haplotypes. These regions are added to the consensus file that is subtracted against for the second haplotype in the hierarchy, and so on (https://github.com/henni164/Pca_pangenome/tree/main/Figure5) [1]. After all regions are assigned hierarchically, those <= 50 Kb were filtered out and the remaining regions were visualized with R package ggplot2 [2].

For the analysis of Australian haplotypes, parent haplotypes hap3 and hap4 were chosen as the first two haplotypes for hierarchical recombination block assignment. Hap5 was third for being divergent from hap3 and hap4, and its recombinant hap26 was placed in fourth position. Finally, hap6 and hap23, were placed in fifth and sixth position to highlight their divergence from other Australian haplotypes. Regions with low non-reference SNP density from these first six haplotypes were assigned across the other Australian haplotypes (hap25, hap7, hap8, hap27, hap28, hap29, hap30, hap31, hap32). For the analysis of US haplotypes, haplotypes from Pca203 were used first as it is likely the oldest isolate in the collection and caused significant epidemics in the 1940s [3], hap1 and hap2 regions were assigned first across USA haplotypes, hap6, and hap23. Shared sequences from the other USA haplotypes were assigned in descending numeric order with hap6 and hap23 in the final positions. Hap9 and hap14 were not included in this set due to their divergence to the other US haplotypes. Instead, they were visualized with hap3, hap4, hap5, and hap26 regions, as they share more sequence with Australian haplotypes.

References

1. Quinlan AR, Hall IM. BEDTools: a flexible suite of utilities for comparing genomic features. Bioinformatics. 2010;26: 841–842. doi:10.1093/bioinformatics/btq033

2. Wickham H. ggplot2: elegant graphics for data analysis. New York: Springer-Verlag; 2016. Available: https://ggplot2.tidyverse.org

3. Stoa T, Swallers C. Keeping up-to-date on oats. NSDU Agric Exp Station Bimonthly Bull. 1950;12
